# Synthesis, antioxidant capacity and aggregation of carotenoid-curcumin conjugates and hybrids

**DOI:** 10.1371/journal.pone.0347640

**Published:** 2026-05-11

**Authors:** Santiago Jijón, Dalma Czett, Katalin Böddi, Gergely Gulyás-Fekete, Veronika Nagy, Anikó Takátsy, György T. Balogh, József Deli, Attila Agócs

**Affiliations:** 1 Department of Biochemistry and Medical Chemistry, Medical School, University of Pécs, Pécs, Hungary; 2 Department of Pharmaceutical Chemistry, Faculty of Pharmaceutical Sciences, Semmelweis University, Budapest, Hungary; 3 Center for Pharmacology and Drug Research & Development, Semmelweis University, Budapest, Hungary; 4 Department of Chemical and Environmental Process Engineering, Budapest University of Technology and Economics, Budapest, Hungary; 5 Department of Pharmacognosy, Faculty of Pharmacy, University of Pécs, Pécs, Hungary; Dr. Dayaram Patel Pharmacy College, INDIA

## Abstract

Both curcumin and carotenoids are good antioxidants and curcumin has a pleiotropic effect additionally. To combine the beneficial properties carotenoid succinates were coupled to curcumin via ester bond. In another approach hemicurcumin was condensed with apocarotenoid aldehydes to produce a hybrid of the carotenoid and the curcuminoid part. Antioxidant activity of these products was determined with the ABTS-TEAC method in ethanol and in isotonic phosphate buffer saline. Covalent coupling of 8’-apo-β-carotenol, β-cryptoxanthin, capsanthin, and lutein to curcumin via succinate ester significantly increased the antioxidant capacity compared to the parent carotenoids or carotenoid succinates. Derivatization of zeaxanthin, nevertheless, did not improve its properties. The direct merging of hemicurcumin with apocarotenals resulted in extended conjugated polyenes with higher antioxidant activities, that, however, seems to be more effected by the number of phenolic moieties than by the number of conjugated double bonds. The open-chain carotenoid end-group can also contribute to a better antioxidant activity. The lipophilic conjugates and hybrids showed aggregation in aqueous media, thus the determined TEAC values in PBS rather characterize the aggregates than the individual molecules. Based on the drug-prediction studies and TEAC values, bisphenolic hybrids of curcumin with 12,12’-diapo-dialdehyde and crocetindial have the best characteristics for being drug candidates, but an appropriate delivery system is necessary.

## Introduction

Carotenoids are well-known antioxidants, their biochemical effects and implications of disease prevention are extensively detailed in a lot of books and articles [1]. The hydroxy carotenoids (lutein, zeaxanthin, β-cryptoxanthin, capsanthin, 8’-apo-β-carotenol) used in this study are among the most studied carotenoids, as well. All can scavenge free radicals and protect against reactive oxygen species (ROS) by diverse mechanisms [2]. Lutein and zeaxanthin can be found in the skin for photoprotection [3]. They can also penetrate the blood-brain barrier to improve cognitive function [4,5], and the blood-retina barrier to protect macula against UV light [6]. Capsanthin is a very good antioxidant *in vitro* [7], that makes it efficient as chemopreventive, antitumor, and anti-inflammatory agent [8,9]. β-cryptoxanthin is a provitamin of vitamin A (retinol) and seems to have some role in bone calcification [10]. Carotenoids or apocarotenoids can participate in certain signal pathways which suggest that there is more to their biological function than their general antioxidant effect [11]. Apocarotenoid are intermediates produced in humans by the oxidation of carotenoids and can exert biochemical functions that were traditionally attributed to their parent carotenoids [12,13].

Curcumin has been known as a therapeutic agent for thousands of years. It has shown to have multiple biological activities such as antioxidant, cardio- and neuroprotective, antidiabetic, antimicrobial, antimalarial, anti-HIV, thrombosuppressive, antitumor and chemopreventive activities. Several studies indicate that curcumin is a classical example of polypharmacology [14], being able to interact and regulate multiple molecular targets. That supports the belief that a wide variety of biochemical and molecular cascades are affected by this compound [15]. Curcumin shows antioxidant activity by scavenging free radicals, quenching singlet oxygen, and acting as a chelating agent. The antioxidant activity of curcumin originates from the phenolic character of the molecule, which is able to donate a hydrogen atom to lipid alkyl or peroxyl radicals [16,17]. This results in the formation of a resonance-stabilized radical with low reactivity, and this way the radical chain reactions are ceased. The chelating of the pro-oxidant ferrous and ferric ions by curcumin makes it an efficient secondary antioxidant [18]. It has been found that the presence of enolate in the solution is important in the radical-scavenging ability of curcumin [19].

Based on the advantageous properties of both carotenoids and curcumin, it seemed a good idea to combine the two molecules in the hope that a powerful antioxidant arises or a molecule with hitherto unknown properties would be produced. Previously, we combined successfully carotenoids with other antioxidants such as flavonoids [20], melatonin [21] or cysteine [22]. Other examples for carotenoid-antioxidant conjugates are a carotenoid-vitamin E glyceride synthetized by Larsen et al. [23] and a bixin-ascorbic acid ester produced enzymatically [24].

We chose two ways for the combination of these two molecules: synthesis of half-carotenoid half-curcumin hybrids by a condensation reaction, and esterification of curcumin with carotenoid succinates.

## Materials and methods

### Chemicals

The carotenoids were isolated from red pepper *Capsicuum anuum* using a well-established procedure [25]. Crude 8’-apo-β-carotenol was freshly prepared from commercially available 8’-apo-β-carotenal (Fluka), because the alcohol is susceptible to oxidation [26]. The carotenoid aldehydes were donated by CaroteNature Gmbh.

All reagents used for synthesis were analytically pure quality and all organic solvents were of HPLC grade. Organic solutions were dried over anhydrous Na_2_SO_4_ and concentrated in vacuo at 40° C (bath temperature). Thin-layer chromatography (TLC) was performed on Kieselgel 60 F_254_ (Merck), and the plates were visualized under UV light. Silica gel 60 PLC plates were purchased from Merck (Merck & Co., Inc., Rahway, NJ USA). For column chromatography Kieselgel 60 (VWR, particle size 0.063–0.200 mm) was used.

For the antioxidant assay the carotenoids or the conjugates were dissolved in dimethyl sulfoxide (DMSO) (VWR International Kft., Hungary). For the Trolox Equivalent Antioxidant Capacity (TEAC) assay the following reagents were used: 2,2′-azino-di-(3-ethylbenzthiazoline sulfonic acid) (Tokyo Chemical Industries, Japan), potassium persulphate (Alfa Aesar), Gibco Dulbecco’s Phosphate-Buffered Saline (DPBS) powder without Ca^2+^ and Mg^2+^ (VWR International Kft., Hungary), trolox (Acros Organics).

### Characterization of the synthesized compounds

Melting points were measured on a Stuart SMP30 apparatus. NMR spectra were recorded with a Bruker Avance III Ascend 500 spectrometer (500/125 MHz for ^1^H/^13^C) in CDCl_3_, except otherwise indicated. Chemical shifts are referenced to the residual solvent signals. Molar masses were obtained by an Autoflex II MALDI instrument (Bruker Daltonics). DHB (dihydroxy benzoic acid) matrix was used for the ionization of the samples. Mass spectra were monitored in positive mode with pulsed ionization (λ = 337 nm; nitrogen laser, maximum pulse rate: 50 Hz). Spectra were measured in reflectron mode using a delayed extraction of 120 nsec. Spectra were the sum of 1000 shots, external calibration has been implemented. Data processing was executed with Flex Analysis software packages (version: 2.4.). The elemental analysis measurements were performed on a Fisons EA 1110 CHNS apparatus. The UV-Vis spectrophotometric measurements were implemented on a Jasco spectrophotometer model V-550 UV/Vis.

### ABTS-TEAC determination

The assay was performed according to a literature process with slight modifications [2,21,27]. The ABTS^•+^ radical cation was produced by reacting 7 mM 2,2′-azino-bis(3-ethylbenzothiazoline-6-sulfonic acid) and 2.45 mM potassium persulfate in water. The stock ABTS^•+^ solution was prepared 12−16 hours before the experiments and stored at room temperature in dark. The absorbance of the ABTS^•+^ solution was set to 0.70 ± 0.05 at 734 nm by a ca. 100-fold dilution with ethanol (96%). Trolox was dissolved in ethanol, the carotenoids and their conjugates in dimethyl sulfoxide (DMSO) to acquire 2.5· 10^−4^ M stock solutions, which were further diluted with 96% ethanol to obtain 1.875 ∙ 10^−4^, 1.25 ∙ 10^−4^, 6.25 ∙ 10^−5^, 3.125 ∙ 10^−5^, and 1.5625 ∙ 10^−5^ M concentrations, respectively. 60 µL portions of these solutions were incubated with 2940 µL of ABTS^•+^ solution at 37 °C for 6 minutes. The final concentrations of the antioxidants were 0, 0.3125, 0.625, 1.25, 2.50, 3.75, 5.00 μM in the reaction mixtures. During the reaction of ABTS^•+^ with the antioxidants the absorbance of the solution decreases. The percentage inhibition of absorbance at 734 nm was calculated as (*A*_0_ - *A*_antioxidant_)/*A*_0_, where A_0_ is the absorbance of the ABTS^•+^ solution and *A*_antioxidant_ is the absorbance measured after the addition of the antioxidant (all corrected for the solvent). The determinations were carried out at each concentration in triplicate. The calculated percentage inhibition values were plotted against the final concentration of the antioxidants. The slopes of the curves were compared with that for trolox, the TEAC value is the ratio of the slopes for the antioxidant and for trolox.

The TEAC values were also determined in isotonic phosphate-buffered saline (PBS) of pH 7.4 (the DMSO stock solutions and the ABTS^•+^ solution were diluted with PBS solution instead of ethanol). The buffer solution was made by dissolving 9.55 g of DPBS powder in distilled water to form 1L of solution (composition: 0.138 M NaCl, 0.0027 M KCl, 0.0081 M Na_2_HPO_4_, 0.0015 M KH_2_PO_4_). In the blank (0 μM), the solutions of the antioxidants were substituted with ethanol, or PBS, respectively. The measurements were performed in a 1 cm optical path quartz cuvette with a 1 nm resolution, in the wavelength range of 260–600 nm. Jasco spectrophotometer model V-730 UV/Vis (Jasco Corporation, Japan) was used for recording the UV-Vis spectra.

### Determination of hydrodynamic diameter of aggregates by dynamic light scattering

The samples were prepared the same way as in the ABTC assay, with the difference that instead of ABTS^•+^ reagent they contained only solvent. Each sample was examined after 6 min incubation time at 37 °C.

The size of the aggregates in the sample dispersions was determined by dynamic light scattering (DLS) photometric measurements, where the hydrodynamic diameter of particles was measured. The number-weighted size distribution indicated one main peak in most cases, the polydispersity index (PDI) was calculated as sd^2^/mean size^2^. For the particle size analysis, a Malvern Zetasizer Nano S (Malvern Panalytical Ltd., Great Malvern, Worcestershire, UK) apparatus was used. The size of the particles was obtained by the average of 11 measurement cycles. The measurements were carried out in autocorrelation mode, and the following parameters were kept constant: scattering angle 173°, attenuator 11 and its factor 0.0146, measurement position 4.65 mm.

### Statistical analysis

All experiments were done in triplicate. Data were expressed as means±SD. After the normality test (Kolmogorov-Smirnov test) and homogeneity of variance test (Levene’s test) for the comparison of the means one way ANOVA with Tukey post-hoc test was calculated if the homogeneity of the variance was assummed. If the homogeneity of the variance was not assummed Welch ANOVA with Games-Howell post-hoc test was implemented by using SPSS 26.0 (SPSS, Chicago, IL, USA). A difference was considered statistically significant at p < 0.05.

## Results and discussion

### Synthesis of hybrids from apocarotenals and hemicurcumin

**Scheme 1:**
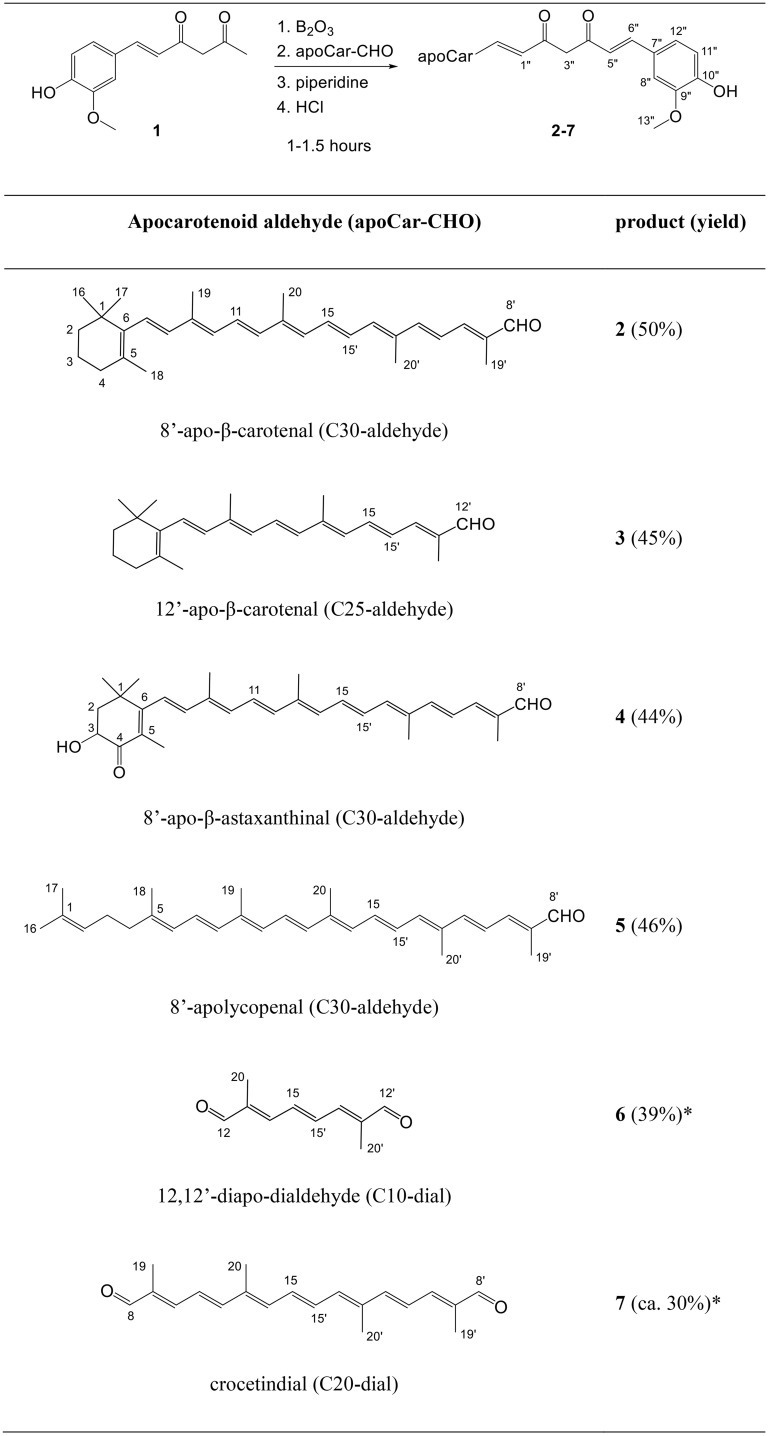
Synthesis of hybrids from apocarotenals and hemicurcumin 1 (HC). *In compounds **6** and **7** both formyl groups were condensed with hemicurcumin.

Curcumin itself can be synthesized via the condensation of vanillin and acetylacetone using a literature procedure [28,29]. This condensation gives first hemicurcumin (**1**, HC, (*E*)-6-(4-hydroxy-3-methoxyphenyl)hex-5-ene-2,4-dione) in bulk amounts as a yellow crystalline powder in a 75% yield. The same articles describe a condensation of hemicurcumin to aromatic aldehydes, as well. Carotenoid aldehydes are, however, not common in nature, they are used usually as intermediates of carotenoid total synthesis. Due to the generous donation of CaroteNature Gmbh. we had access to some aldehydes and could use them in our experiments (Scheme 1).

Based on the literature data 10–15 min should be enough for the formation of the boron complex with hemicurcumin, however, TLC showed only an ~ 80% conversion of the apocarotenal even added to the reaction mixture after 30 min. We could not reach higher yields or conversions even if the hemicurcumin was applied in a 3–4-fold excess, probably because of the instability of the apocarotenoid aldehyde. On the other hand reaction time was restricted to approx. 1 hour and temperature was lowered to 70 °C to avoid decomposition of the carotenoids and the products. Byproducts appeared on TLC either with very high or very low mobility, as well. Still, yields were moderate or quite good considering that at least two column and/or PLC chromatographies and a crystallization were needed to obtain pure products. The crocetindial hybrid (**7**) could not be obtained in pure form even after several chromatographies as it possessed the same retention as HC, its 85–90% purity was assessed from the NMR spectra. As it could be clearly identified by NMR and MS, it was included in the antioxidant studies. The products have deep colors (see S6 Fig in S1 File) because the length of the conjugated polyene chain increased considerably with the conjugation. That was also expected to exhibit higher antioxidant capacity.

### Synthesis of carotenoid-curcumin mixed esters

**Scheme 2:**
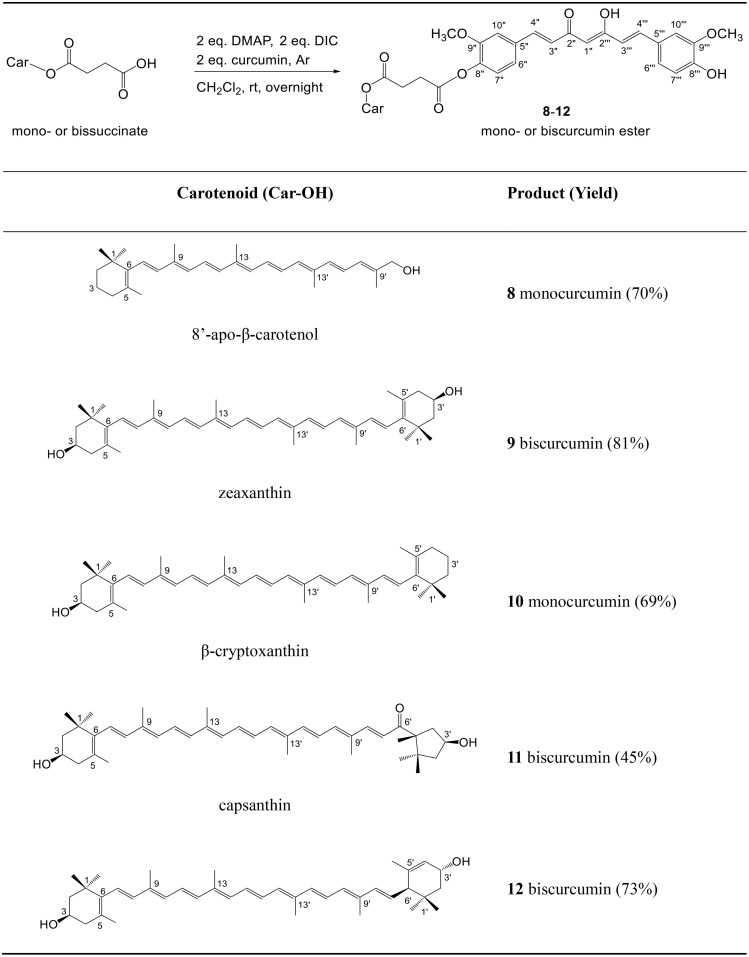
Synthesis of curcumin conjugates from carotenoid mono- and bissuccinates.

To test the possible synergism between carotenoids and curcumin we planned the preparation of conjugates of these two. The easiest way to achieve this was to use carotenoid succinates, which can be efficiently synthetized from hydroxy carotenoids [21]. Steglich-type esterification was used to couple the succinates to curcumin, the latter was in excess in all reactions (Scheme 2). Instead of dicyclohexylcarbodiimide (DCC) the more convenient diisopropylcarbodiimide (DIC) was used for coupling. As products usually have similar R_f_ values as curcumin, separation was possible only on preparative layers (PLC) and crystallization was also needed.

### In silico physicochemical and early ADME (absorption, distribution, metabolism, and excretion) characterization of carotenoid-curcumin derivatives

The physicochemical properties of the newly synthesized carotenoid-curcumin derivatives were initially characterized according to the Lipinski rule of five (Ro5) for drug-likeness [30]. As expected in the case of such hybrid molecules, the conjugates exceed the limits defined by the Ro5 criteria system in several of their parameters. Thus, based on the data in Table 1, it can be seen that M_w_ > 500, logP>>5 (with the exception of compound **6**), and HBA > 10 for the compounds **9**, **11** and **12**. The polar surface area of the new compounds does not significantly exceed the 120 Å^2^ value for oral absorption (exception of **9**, **11** and **12**), while the 90 Å^2^ limit related to BBB permeability [31] is met only by compounds **2**, **3** and **5**. Regarding the BCS system [32], the estimated solubility and Caco2 permeability of the conjugates are particularly weak in terms of medicinal chemistry, so all conjugates except compound **6** can be classified in the BCS IV class. Similarly, looking at the in silico logBB values, a slight CNS tissue saturation is expected for only compound **6** and **7**, the BBB penetration of the other derivatives is negligible. Overall, based on the in silico early ADME data, **6** and **7** conjugates can be selected as primary test candidates, but it must be emphasized that in the case of these two carotenoid-curcumin derivatives, it is necessary to develop the appropriate formulation to treat the expected reduced solubility.

**Table 1:** Predicted (using ACD/Labs Percepta Release 2021.2.1 (Build 3525, accessed on 17 Dec 2021), www.acdlabs.com/products/percepta/) physicochemical and early ADME profile of investigated curcumin and its derivatives.

Predicted parameters										
Compound	M_w_		Acidic p*K*_*a1/a2*_		log*P/* logD_7.4_^a^	HBD/HBA	TPSA Å^2^	Aq.Sol.^b^ mg/ml	Caco-2 permeability P_e_	logBB [40]
									10^−6^ cm/s	
									[39]	
**2**	632.9		8.4/ 10.0		10.4/ 10.3	02-Apr	66.8	2·10^-6^	0.2	−0.87
**3**	566.8		8.4/ 10.0		9.3/ 9.2	02-Apr	66.8	8·10^-6^	0.3	−0.63
**4**	662.9		8.4/ 10.0		7.3/ 7.2	03-Jun	104.1	2·10^-7^	1	−0.16
**5**	632.9		8.4/ 10.0		10.4/ 10.3	02-Apr	66.8	5·10^-7^	0.2	−0.8
**6**	596.7		8.0/ 8.7		4.2/ 4.1	04-Aug	133.5	3·10^-6^	11.2	0.2
**7**	728.9		8.0/ 8.7		7.2/ 7.1	04-Aug	133.5	9·10^-9^	0.5	0.05
**8**	869.1		8.1/ 10.1		12.6/ 12.5	01-Sep	125.4	5·10^-8^	0.1	−2
**9**	1469.8		8.0/ 8.6		18.1/ 18.0	Apr-18	257.2	Insoluble	0.1	−2
**10**	1003.3			8.1/ 10.1	16.0/ 15.9	01-Sep	125.4	Insoluble	0.1	−2
**11**	1485.8			7.8/ 8.4	16.4/ 16.2	Feb-19	267.9	Insoluble	0.1	−2
**12**	1469.8	8.0/ 8.6			17.3/ 17.2	Apr-18	257.2	Insoluble	0.1	−2
Curcumin	368.4	8.4/ 9.7			2.6/ 2.6	03-Jun	96.2	8·10^-2^	46	0.04
HC	234.3		8.8/ 10.1		1.3/ 1.2	01-Apr	63.6	1.67	73.8	−0.17

^a^Calculated using logP/logD_7.4_ (Consensus and pK_*a*_ (Classic) settings within Percepta package. ^b^ aqueous solubility: logS_pH6.5_ (at intestinal conditions) using Drug Profiler unit of Percepta Package. HC: hemicurcumin.

Delivery methods for curcumin [33] or carotenoids [34] in the form of nanoencapsulation have been elaborated in the last 10–15 years and resulted in higher absorption, so these methods could be used in our case, as well. Previously, we successfully integrated carotenoids and derivatives into lecithin-based liposomes but any previous antioxidant effect disappeared so we abandoned this approximation. Under certain conditions cyclodextrin-carotenoid complexes can serve as potent delivery systems as we recently showed in an eye model [35]. Very recently, some new delivery technologies emerged such as niosomes [36] and aspasomes [37], which could probably be used for carotenoid encapsulation, as well. Nevertheless the above ADME tests or PAMPA (Parallel Artificial Membrane Permeability Assay) measurements cannot predict facilitated diffusion, which is the case in the intestinal absorption of carotenoids that are absorbed mostly in micelles in cases rather efficiently, without any delivery system [38].

### Antioxidant properties

For the description of the *in vitro* antioxidant behavior of the newly synthetized derivatives ABTS-TEAC assays were made to estimate changes in the antioxidant capacity compared to the parent (apo)carotenoids, and underivatized curcumin or hemicurcumin. Trolox is the water-soluble analogue of vitamin E and is generally used as reference molecule in antioxidant studies. The measurements were performed in ethanol and in phosphate-buffered saline (PBS). The TEAC values from ethanol characterize the inherent antioxidant property of the molecules, while those from PBS may depict better the behavior of molecules among physiological conditions. The connections between solvent, TEAC values and aggregation have recently been investigated in detail [2]. For detailed description of the statistical analysis see S1-S4 Tables in S1 File.

**Fig 1:**
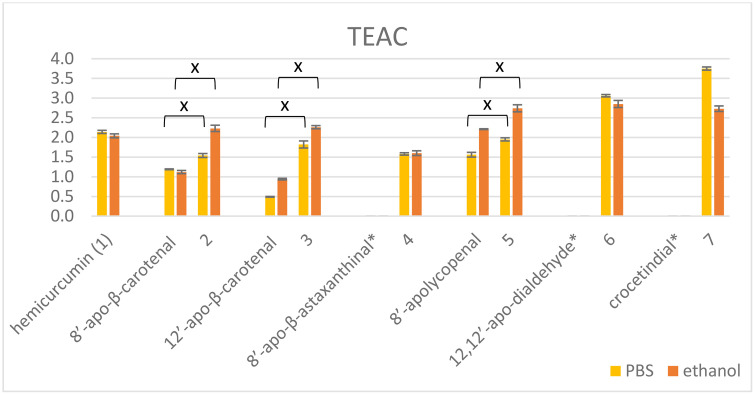
ABTS-TEAC values of hemicurcumin and its hybrids compared to parent apocarotenals in PBS (yellow) and ethanol (orange). *TEAC was not possible to determine. The ‘x’ indicates statistically significant difference according to ANOVA and Tukey’s post hoc or Games-Howell post hoc test (p < 0.05). **7** contained ~10% HC.

Beside the TEAC values of hemicurcumin (HC) and its hybrids with apocarotenals, TEAC for the parent apocarotenals were also calculated. In some cases, the parent aldehydes were unstable, and the determination of TEAC was not possible among the standard conditions (Fig 1). Hybrids and conjugates were stable enough to do the assays with them and their stock solutions in DMSO could be kept for 2–3 weeks at −20 ºC without noticeable decomposition. The hybrid **7** contained ~15% HC, its TEAC values are considered as an estimation.

Three hybrids, **5** with 8’-apolycopenal, **6** with 12,12’-diapo-dialdehyde, and **7** with crocetindial showed significantly higher antioxidant activity than hemicurcumin in ethanol, and only **6** and **7** in PBS. Comparing the structure of the hybrids, compounds with two hemicurcumin moieties had unambiguously the best TEAC values in PBS. In ethanol, however, the open-chain lycopene derivative (**5**) also exhibited an increased antioxidant capacity. The TEAC value of **4** (HC + 8’-apo-β-astaxanthinal) fell short of **6** (2 HC + 12,12’-diapo-dialdehyde), although both contains the same high number of conjugated double bonds (Table 2). Similarly, compound **2** (HC + 8’-β-apocarotenal) and **5** (HC + 8’-apolycopenal) both have 16 conjugated double bonds and only one hemicurcumin moiety, the only difference is the cyclic (**2**) or open-chain (**5**) structure of the carotenoid end-group. Compounds **2** (HC + 8’-β-apocarotenal), **3** (HC + 8’-apolycopenal), and **4** (HC + 8’-apo-β-astaxanthinal) gave very similar TEAC values in spite of having different numbers of conjugated double bonds.

**Table 2:** Number of conjugated double bonds and phenolic moieties in hemicurcumin and in its hybrids with apocarotenals.

compound	conjugated double bonds		phenolic moieties
**1** (HC)	6	= 6	1
**2** (HC + 8’-β-apocarotenal)	7 + 9	= 16	1
**3** (HC + 12’-apo-β-carotenal)	7 + 7	= 14	1
**4** (HC + 8’-apo-β-astaxanthinal)	7 + 10	= 17	1
**5** (HC + 8’-apolycopenal)	7 + 9	= 16	1
**6** (2 HC + 12,12’-diapo-dialdehyde)	2 x 7 + 3	= 17	2
**7** (2 HC + crocetindial)	2 x 7 + 7	= 21	2

Considering the above, the antioxidant behavior against ABTS^•+^ seems to be more effected by the number of phenolic moieties than by the number of conjugated double bonds. However, the cyclic end-group of the carotenoid moiety represents a steric hindrance and prevents the perfect overlap of *p* orbitals for π bonds C-5,6 and C-7,8. [41]. Thus, the open-chain carotenoid end-group can also contribute to a better antioxidant activity.

**Fig 2:**
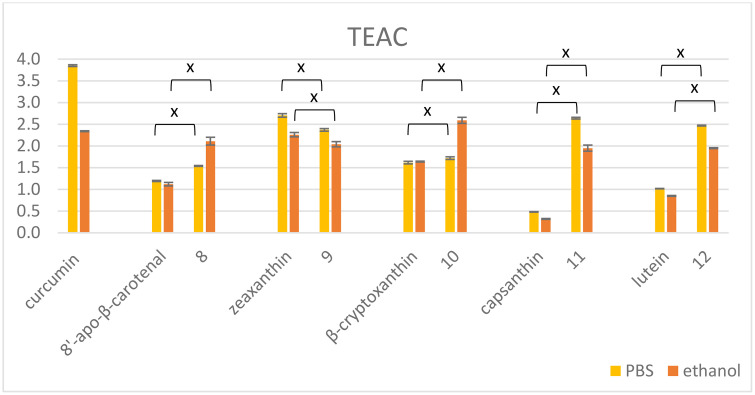
ABTS-TEAC values of curcumin and its ester conjugates with carotenoids compared to parent carotenoids in PBS (yellow) and ethanol (orange). The ‘x’ indicates statistically significant difference according to ANOVA and Tukey’s post hoc or Games-Howel post hoc test (p < 0.05).

In the case of carotenoid succinate-curcumin esters (**8**–**12**) the TEAC values were quite similar to each other and that of curcumin in ethanol, independently on the number of curcumin moieties (Fig 2). With the exception of zeaxanthin derivative **9**, all the conjugates surpassed the parent carotenoids in antioxidant capacity. In PBS curcumin showed a much higher TEAC value than the derivatives, and the conjugates having the same number of curcumin moieties (monocurcumins **8**, **10**, and biscurcumins **9**, **11**, **12**) behaved in a similar manner to each other.

Comparing the TEAC values of the hybrids and the ester conjugates, the direct coupling with the extension of the conjugated polyene system seems to result in more potent antioxidants. However, the more phenolic moieties are present, the higher the antioxidant capacity.

### Aggregation studies

**Fig 3:**
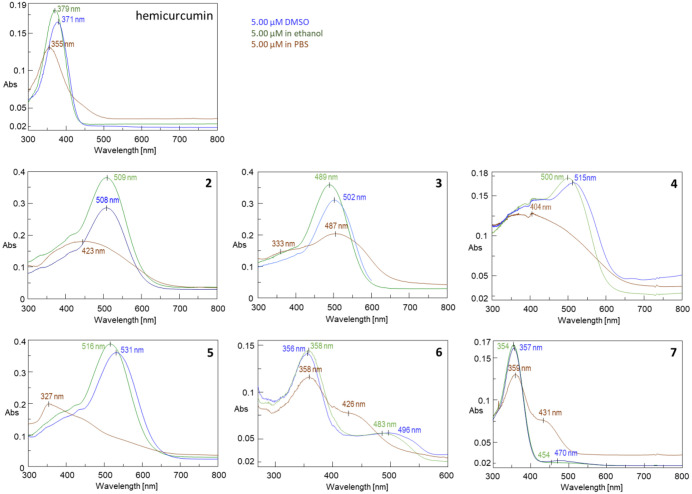
UV-vis spectra of hemicurcumin and its hybrids with apocarotenals recorded in DMSO (blue), ethanol (green) and PBS (red) at 5 μM concentration.

As the synthesized derivatives of curcumin are hydrophobic compounds, they were expected to aggregate in aqueous solution. To examine this behavior samples were prepared the same way as in the ABTS experiments, with the difference that instead of ABTS reagent only solvent (DMSO, 96% ethanol, or PBS) was used. These samples were examined by UV-vis and dynamic light scattering photometries (Fig 3).

The aggregation of hemicurcumin-apocarotenal hybrids was studied by comparing the UV-vis spectra in different solvents. In pure DMSO and ethanol the spectra showed that aggregation did not occur. A decrease in the intensities and change of the absorption wavelength (λ_max_) in PBS compared to that in pure DMSO clearly indicated the formation of aggregates. Thus, the determined TEAC values in PBS rather characterize the aggregates than the individual molecules [2]. An intense hypsochromic shift was also observed in the case of **2**, **4** and **5**. Such changes in the UV-vis spectrum generally suggest the formation of H-type (card pack) assemblies of molecules [42]. All these hybrids contain at least one phenolic OH capable of hydrogen bonding, which facilitates the formation H-type aggregates [43]. Nevertheless, compounds **3**, **6** and **7** rather seems to form J-type (head-to-tail) or mixed type aggregates.

**Fig 4:**
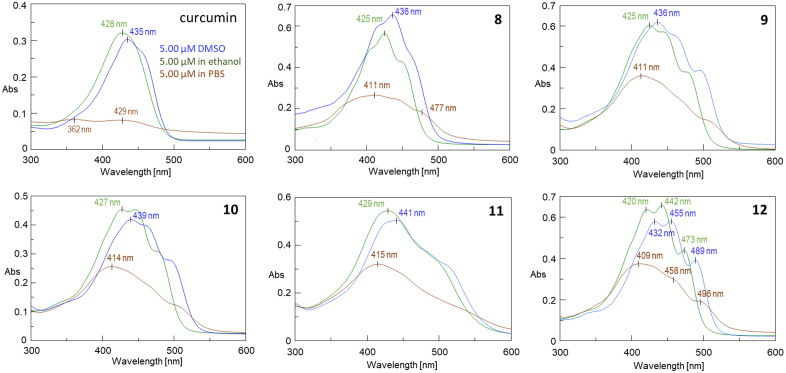
UV-vis spectra of curcumin and its conjugates with carotenoids recorded in DMSO (blue), ethanol (green) and PBS (red) at 5 μM concentration.

The curcumin conjugates with carotenoid succinates behaved similarly, however, the changes in the UV spectra indicated mixed type aggregate formation (Fig 4).

In these compounds the succinate linker makes the molecules more flexible. With the exception of compound **12**, the UV spectra of the new compounds at different concentrations in PBS showed very similar shapes (see S1-S5 Fig in S1 File) implying similar type of aggregations. However, UV-vis spectrophotometry alone is not sufficient to establish the exact type of aggregates.

**Fig 5:**
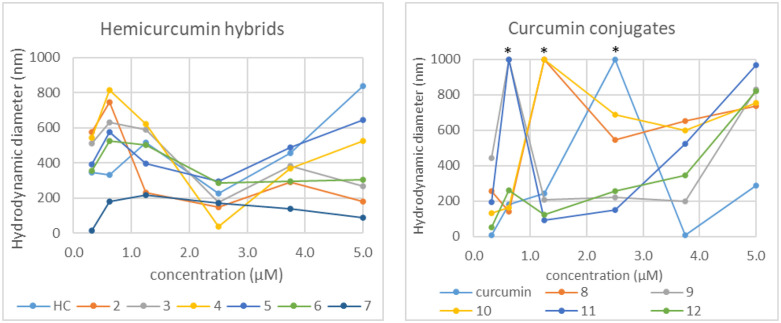
Hydrodynamic diameters of the synthesized derivatives determined by DLS in PBS. *The particles were too large for the determination.

The hydrodynamic diameter of the aggregates was determined by dynamic light scattering (DLS) photometry (Fig 5).

The hemicurcumin hybrids behaved very similarly to hemicurcumin forming aggregates of ca. 200–800 nm. An intriguing concentration dependence was observed, at 2.5 μM concentrations almost all compounds gave a minimum particle size. The only exception was **7** (2 HC + crocetindial), that formed small aggregates of similar size (ca. 100–200 nm) almost independently on the concentration. That can also explain the highest TEAC value of this compound in PBS.

The curcumin-carotenoid conjugates showed two patterns: compounds **8** and **10** above 1 μM concentrations formed aggregates higher than 600 nm, while **9**, **11** and **12** gave smaller particles, typically below 400 nm diameter. That also correlates to the higher TEAC values of **9**, **11** and **12**. Interestingly, at low concentrations such as 0.625 or 1.25 μM the conjugates formed huge clumps, which were out of the range of determination by DLS.

Scanning electron microscopy (SEM) was used to study the morphology of the formed particles, and for that the dispersions were freeze-dried. However, the samples from PBS were not suitable for SEM analysis, since the solid material left after freeze-drying consisted mainly of huge crystals of the salts of the buffer, the carotenoid aggregates were very difficult to find among them. SEM pictures could be made for dispersions from pure water, but the aggregation from this solvent was rather different from that in PBS (different particle-size and TEAC were determined). As the dispersions in PBS were prepared for immediate ABTS experiments, the colloidal stability (zeta-potential) was not examined.

## Conclusions

With the exception of zeaxanthin, covalent coupling of all carotenoids to curcumin significantly improved the antioxidant capacity compared to the parent carotenoids or carotenoid succinates. Nevertheless, the direct merging of hemicurcumin with apocarotenals resulted in extended conjugated polyenes with higher antioxidant activities. Based on the drug-prediction studies and TEAC values, bisphenolic compounds **6** and **7** have the best characteristics as promising molecular scaffolds, but an appropriate delivery system is necessary for further biological studies.

## Supporting information

S1 File Fig S1-S5: UV-spectra of the hybrids and conjugates in ethanol and PBS at different concentrations. Fig S6: Crystals of 8’-apo-β-carotenal-hemicurcumin hybrid (2). Table S1-S4: Statistical evaluation of the antioxidant measurements.
(PDF)

S1 Appendix Experimental Section. (PDF)

## References

[1] Rodriguez-Concepcion M, Avalos J, Bonet ML, Boronat A, Gomez-Gomez L, Hornero-Mendez D, et al. A global perspective on carotenoids: Metabolism, biotechnology, and benefits for nutrition and health. Prog Lipid Res. 2018;70:62–93. doi: 10.1016/j.plipres.2018.04.004 29679619

[2] Czett D, Nagy V, Kurtán T, Király SB, Szabó P, Agócs A, et al. Effect of aggregation behaviour on the antioxidant capacity of carotenoids. Journal of Molecular Liquids. 2025;421:126870. doi: 10.1016/j.molliq.2025.126870

[3] Roberts RL, Green J, Lewis B. Lutein and zeaxanthin in eye and skin health. Clin Dermatol. 2009;27(2):195–201. doi: 10.1016/j.clindermatol.2008.01.011 19168000

[4] Hammond BR Jr, Miller LS, Bello MO, Lindbergh CA, Mewborn C, Renzi-Hammond LM. Effects of Lutein/Zeaxanthin Supplementation on the Cognitive Function of Community Dwelling Older Adults: A Randomized, Double-Masked, Placebo-Controlled Trial. Front Aging Neurosci. 2017;9:254. doi: 10.3389/fnagi.2017.00254 28824416 PMC5540884

[5] Renzi-Hammond LM, Bovier ER, Fletcher LM, Miller LS, Mewborn CM. Effects of a lutein and zeaxanthin intervention on cognitive function: A randomized, double-masked, placebo-controlled trial of younger healthy adults. Nutrients. 2017;9. 10.3390/nu9111246 PMC5707718 29135938

[6] Bernstein PS, Li B, Vachali PP, Gorusupudi A, Shyam R, Henriksen BS, et al. Lutein, zeaxanthin, and meso-zeaxanthin: The basic and clinical science underlying carotenoid-based nutritional interventions against ocular disease. Prog Retin Eye Res. 2016;50:34–66. doi: 10.1016/j.preteyeres.2015.10.003 26541886 PMC4698241

[7] Pérez-Gálvez A, Mínguez-Mosquera MI. Structure-reactivity relationship in the oxidation of carotenoid pigments of the pepper (Capsicum annuum L.). J Agric Food Chem. 2001;49(10):4864–9. doi: 10.1021/jf010547c 11600036

[8] Kennedy LE, Abraham A, Kulkarni G, Shettigar N, Dave T, Kulkarni M. Capsanthin, a Plant-Derived Xanthophyll: a Review of Pharmacology and Delivery Strategies. AAPS PharmSciTech. 2021;22(5):203. doi: 10.1208/s12249-021-02065-z 34244867

[9] Murillo EN, Agócs A, Deli J. Carotenoids with κ-end group. In: Yamaguchi M, editor. Carotenoids: Food Sources, Production and Health Benefits. Nova Science Publishers Inc. 2013. p. 49–78.

[10] Burri BJ, La Frano MR, Zhu C. Absorption, metabolism, and functions of β-cryptoxanthin. Nutr Rev. 2016;74(2):69–82. doi: 10.1093/nutrit/nuv064 26747887 PMC4892306

[11] Stahl W, Ale-Agha N, Polidori MC. Non-antioxidant properties of carotenoids. Biol Chem. 2002;383(3–4):553–8. doi: 10.1515/BC.2002.056 12033443

[12] Chung J, Koo K, Lian F, Hu KQ, Ernst H, Wang X-D. Apo-10’-lycopenoic acid, a lycopene metabolite, increases sirtuin 1 mRNA and protein levels and decreases hepatic fat accumulation in ob/ob mice. J Nutr. 2012;142(3):405–10. doi: 10.3945/jn.111.150052 22259190 PMC3278264

[13] Ip BC, Liu C, Lichtenstein AH, von Lintig J, Wang XD. Lycopene and Apo-10 ‘-lycopenoic Acid Have Differential Mechanisms of Protection against Hepatic Steatosis in Beta-Carotene-9 ‘,10 ‘-Oxygenase Knockout Male Mice. Journal of Nutrition. 2015;145:268–76. 25644347 10.3945/jn.114.200238 PMC4304024

[14] Jalencas X, Mestres J. On the origins of drug polypharmacology. Med Chem Commun. 2013;4(1):80–7. doi: 10.1039/c2md20242e

[15] Deguchi A. Curcumin targets in inflammation and cancer. Endocr Metab Immune Disord Drug Targets. 2015;15(2):88–96. doi: 10.2174/1871530315666150316120458 25772169

[16] Jayaprakasha GK, Jaganmohan Rao L, Sakariah KK. Antioxidant activities of curcumin, demethoxycurcumin and bisdemethoxycurcumin. Food Chemistry. 2006;98(4):720–4. doi: 10.1016/j.foodchem.2005.06.037

[17] Barclay LR, Vinqvist MR, Mukai K, Goto H, Hashimoto Y, Tokunaga A, et al. On the antioxidant mechanism of curcumin: classical methods are needed to determine antioxidant mechanism and activity. Org Lett. 2000;2(18):2841–3. doi: 10.1021/ol000173t 10964379

[18] Ak T, Gülçin I. Antioxidant and radical scavenging properties of curcumin. Chem Biol Interact. 2008;174(1):27–37. doi: 10.1016/j.cbi.2008.05.003 18547552

[19] Zheng B, McClements DJ. Formulation of More Efficacious Curcumin Delivery Systems Using Colloid Science: Enhanced Solubility, Stability, and Bioavailability. Molecules. 2020;25(12):2791. doi: 10.3390/molecules25122791 32560351 PMC7357038

[20] Línzembold I, Czett D, Böddi K, Kurtán T, Király SB, Gulyás-Fekete G, et al. Study on the Synthesis, Antioxidant Properties, and Self-Assembly of Carotenoid-Flavonoid Conjugates. Molecules. 2020;25(3):636. doi: 10.3390/molecules25030636 32024181 PMC7038153

[21] Czett D, Böddi K, Nagy V, Takátsy A, Deli J, Tone P, et al. Synthesis, Pharmacokinetic Characterization and Antioxidant Capacity of Carotenoid Succinates and Their Melatonin Conjugates. Molecules. 2022;27(15):4822. doi: 10.3390/molecules27154822 35956776 PMC9369794

[22] Zand A, Agócs A, Deli J, Nagy V. Synthesis of carotenoid-cysteine conjugates. Acta Biochim Pol. 2012;59(1):149–50. doi: 10.18388/abp.2012_2193 22428132

[23] Larsen E, Abendroth J, Partali V, Schulz B, Sliwka H-R, Quartey EGK. Combination of Vitamin E with a Carotenoid:α-Tocopherol and Trolox Linked toβ-Apo-8′-carotenoic Acid. Chem Eur J. 1998;4(1):113–7. doi: 10.1002/(sici)1521-3765(199801)4:1<113::aid-chem113>3.0.co;2-q

[24] Humeau C, Rovel B, Girardin M. Enzymatic esterification of bixin by l-ascorbic acid. Biotechnology Letters. 2000;22(2):165–8. doi: 10.1023/a:1005665913685

[25] Deli J, Molnár P, Matus Z, Tóth G. Carotenoid composition in the fruits of red paprika (Capsicum annuum var. lycopersiciforme rubrum) during ripening; biosynthesis of carotenoids in red paprika. J Agric Food Chem. 2001;49(3):1517–23. doi: 10.1021/jf000958d 11312889

[26] Agócs A, Bokor É, Takátsy A, Lóránd T, Deli J, Somsák L, et al. Synthesis of carotenoid-monosaccharide conjugates via azide–alkyne click-reaction. Tetrahedron. 2017;73(5):519–26. doi: 10.1016/j.tet.2016.12.035

[27] van den Berg R, Haenen GRMM, van den Berg H, Bast A. Applicability of an improved Trolox equivalent antioxidant capacity (TEAC) assay for evaluation of antioxidant capacity measurements of mixtures. Food Chemistry. 1999;66(4):511–7. doi: 10.1016/s0308-8146(99)00089-8

[28] Masuda T, Matsumura H, Oyama Y, Takeda Y, Jitoe A, Kida A, et al. Synthesis of (+/-)-cassumunins A and B, new curcuminoid antioxidants having protective activity of the living cell against oxidative damage. J Nat Prod. 1998;61(5):609–13. doi: 10.1021/np970555g 9599258

[29] Dai YH, He YZ, Wang Y, Jiang HF, Li ZF. A concise total synthesis of (/-)-cassumunin C. Synthesis-Stuttgart. 2014;46:3041–6.

[30] Lipinski CA. Lead- and drug-like compounds: the rule-of-five revolution. Drug Discov Today Technol. 2004;1(4):337–41. doi: 10.1016/j.ddtec.2004.11.007 24981612

[31] Wager TT, Hou X, Verhoest PR, Villalobos A. Central Nervous System Multiparameter Optimization Desirability: Application in Drug Discovery. ACS Chem Neurosci. 2016;7(6):767–75. doi: 10.1021/acschemneuro.6b00029 26991242

[32] Amidon GL, Lennernäs H, Shah VP, Crison JR. A theoretical basis for a biopharmaceutic drug classification: the correlation of in vitro drug product dissolution and in vivo bioavailability. Pharm Res. 1995;12(3):413–20. doi: 10.1023/a:1016212804288 7617530

[33] Sunoqrot S, Abu Shalhoob M, Jarrar Y, Hammad AM, Al-Ameer HJ, Al-Awaida W. Nanoencapsulated Curcumin Mitigates Liver Injury and Drug-Metabolizing Enzymes Induction in Diclofenac-Treated Mice. ACS Omega. 2024;9(7):7881–90. doi: 10.1021/acsomega.3c07602 38405487 PMC10882592

[34] Dos Santos PP, Andrade L de A, Flôres SH, Rios A de O. Nanoencapsulation of carotenoids: a focus on different delivery systems and evaluation parameters. J Food Sci Technol. 2018;55(10):3851–60. doi: 10.1007/s13197-018-3316-6 30228383 PMC6133860

[35] Angi R, Kalóczkai AJ, Kovács A, Marton A, Bárdos V, Dormán P, et al. Harnessing cyclodextrins for enhanced ocular delivery of carotenoid derivatives: From development to ex vivo characterization. Carbohydrate Polymer Technologies and Applications. 2025;9:100718. doi: 10.1016/j.carpta.2025.100718

[36] Liga S, Paul C, Moacă E-A, Péter F. Niosomes: Composition, Formulation Techniques, and Recent Progress as Delivery Systems in Cancer Therapy. Pharmaceutics. 2024;16(2):223. doi: 10.3390/pharmaceutics16020223 38399277 PMC10892933

[37] Hozan F, Uslu EN, Karatoprak GŞ, Yücel Ç. Baicalein-Loaded Aspasomal Formulations: Development, Characterization and Evaluation of Antioxidant and Anti-Inflammatory Effects. J Pharm Innov. 2025;20(2). doi: 10.1007/s12247-025-09979-2

[38] Reboul E. Mechanisms of Carotenoid Intestinal Absorption: Where Do We Stand? Nutrients 11. 2019. 10.3390/nu11040838 PMC6520933 31013870

[39] Lanevskij K, Didziapetris R. Physicochemical QSAR Analysis of Passive Permeability Across Caco-2 Monolayers. J Pharm Sci. 2019;108(1):78–86. doi: 10.1016/j.xphs.2018.10.006 30321548

[40] Lanevskij K, Dapkunas J, Juska L, Japertas P, Didziapetris R. QSAR analysis of blood-brain distribution: the influence of plasma and brain tissue binding. J Pharm Sci. 2011;100(6):2147–60. doi: 10.1002/jps.22442 21271563

[41] Miller NJ, Sampson J, Candeias LP, Bramley PM, Rice-Evans CA. Antioxidant activities of carotenes and xanthophylls. FEBS Lett. 1996;384(3):240–2. doi: 10.1016/0014-5793(96)00323-7 8617362

[42] Hestand NJ, Spano FC. Expanded theory of H- and J-molecular aggregates: The effects of vibronic coupling and intermolecular charge transfer. Chemical Reviews. 2018;118:7069–163. 29664617 10.1021/acs.chemrev.7b00581

[43] Simonyi M, Bikádi Z, Zsila F, Deli J. Supramolecular exciton chirality of carotenoid aggregates. Chirality. 2003;15(8):680–98. doi: 10.1002/chir.10282 12923806

