# Supplementary material for: Synthesis, antioxidant capacity and aggregation of carotenoid-curcumin conjugates and hybrids
Source: PLoS One. 2026 May 11;21(5):e0347640. doi: 10.1371/journal.pone.0347640 (PMC13160358; doi:10.1371/journal.pone.0347640)
Supplement: S1 File — Fig S1-S5: UV-spectra of the hybrids and conjugates in ethanol and PBS at different concentrations. Fig S6: Crystals of 8’-apo-β-carotenal-hemicurcumin hybrid (2). Table S1-S4: Statistical evaluation of the antioxidant measurements. (PDF) [file pone.0347640.s001.docx]

Supplementary material for

**Synthesis, antioxidant capacity and aggregation of carotenoid-curcumin conjugates and hybrids**

Santiago Jijón^1^, Dalma Czett^1^, Katalin Böddi^1^, Gergely Gulyás-Fekete^1^, Veronika Nagy^1^, Anikó Takátsy^1^, György T. Balogh^2^, József Deli^1,3^, Attila Agócs^1,^*

^1^ Department of Biochemistry and Medical Chemistry, Medical School, University of Pécs, Hungary

^2^ Department of Pharmaceutical Chemistry, Faculty of Pharmaceutical Sciences, Semmelweis University, Budapest, Hungary

^3^ Department of Pharmacognosy, Faculty of Pharmacy, University of Pécs, Hungary

^*^ Corresponding author

E-mail: attila.agocs@aok.pte.hu (AA)

**1. UV-spectra of the hybrids and conjugates in ethanol and PBS at different concentrations**

**2. Statistical evaluation of the antioxidant measurements**

**3. A photo of the shiny, dark curcumin-carotenoid hybrid crystals**

**1. UV-spectra of the hybrids and conjugates in ethanol and PBS at different concentrations**


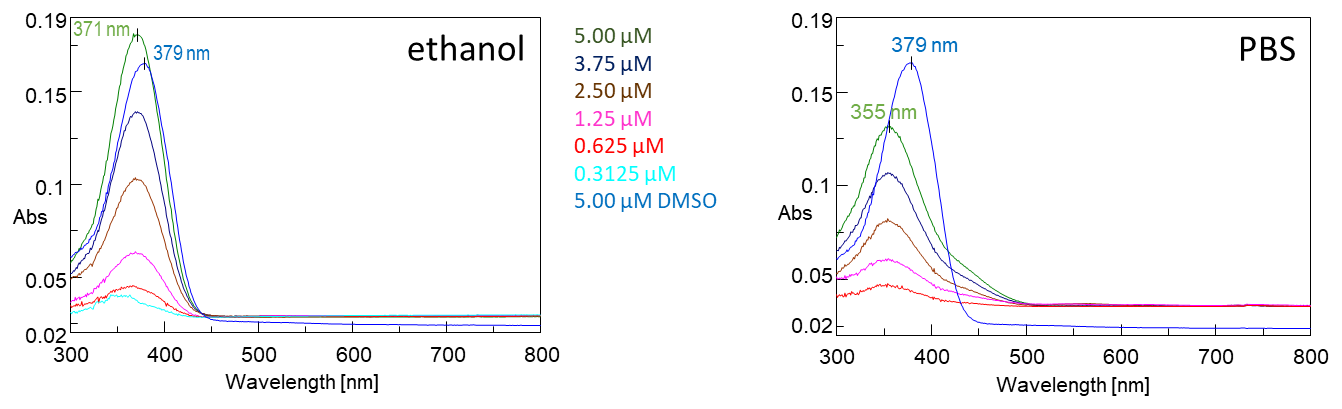


**Fig S1. UV-spectra of hemicurcumin (1) in ethanol and PBS**


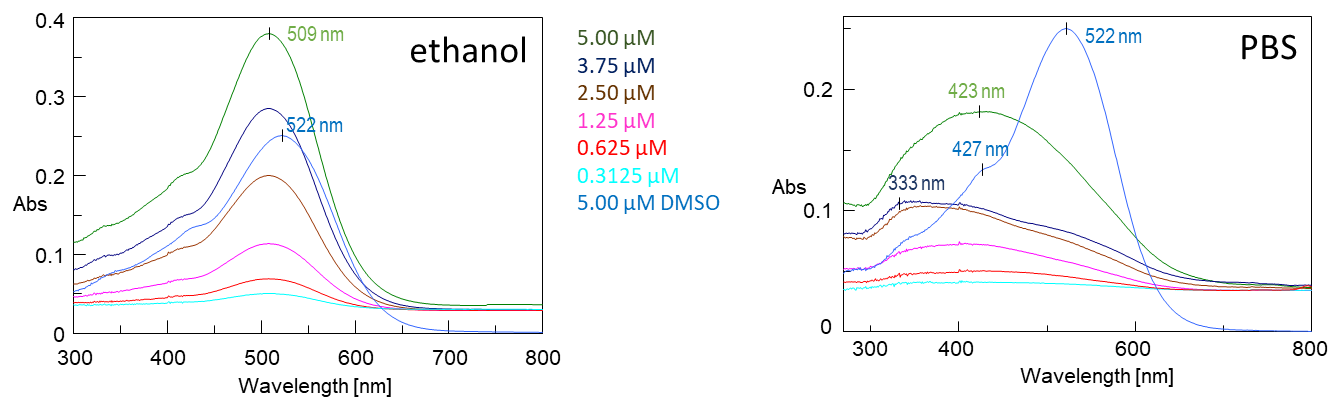


**Fig S2. UV-spectra of 2 (8’-apo-β-carotenal + HC) in ethanol and PBS**

**
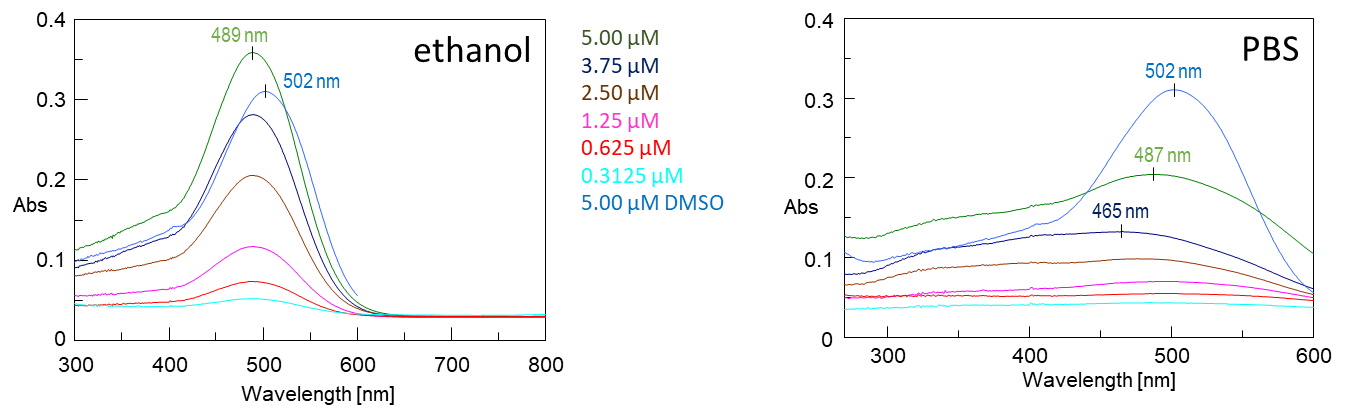
**

**Fig S3. UV-spectra of 3 (12’-apo-β-carotenal + HC) in ethanol and PBS**

**
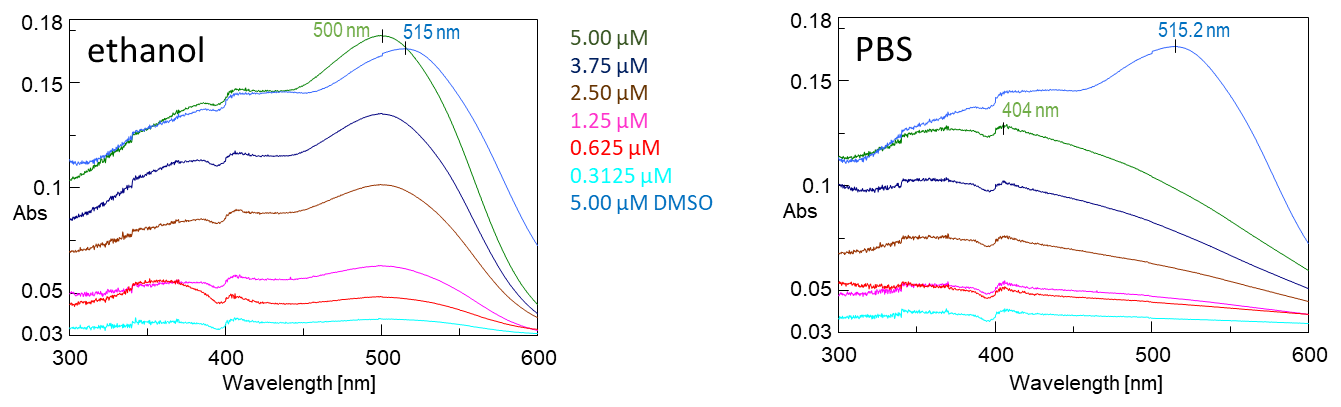
**

**Fig S4. UV-spectra of 4 (8’-apo-β-astaxanthinal + HC) in ethanol and PBS**

**
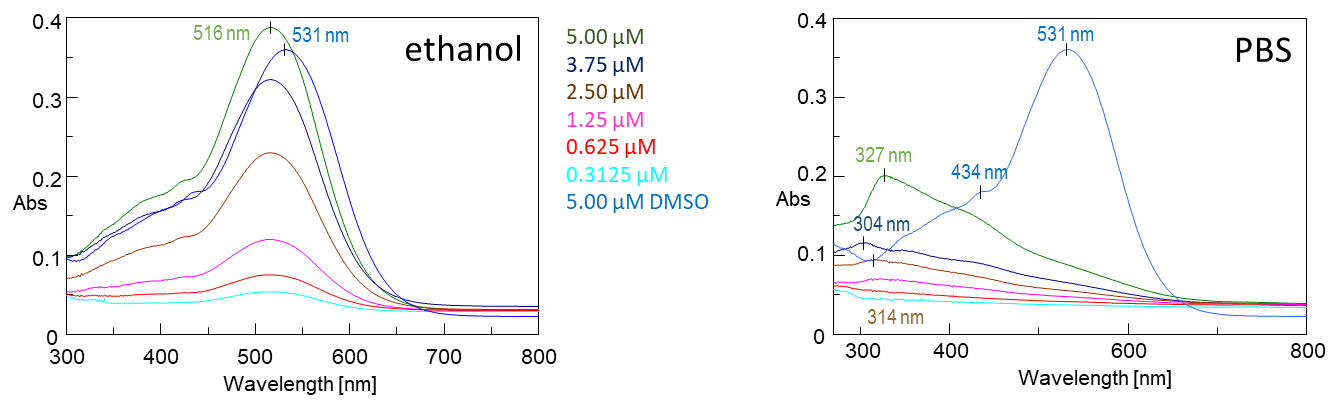
**

**Fig S5. UV-spectra of 5 (8’-apolycopenal + HC) in ethanol and PBS**

**2. Statistical evaluation of the antioxidant measurements**

**Table S1.** Normality test

| compound | ethanol | PBS |
| --- | --- | --- |
| curcumin | 0.800495 | 0.022737 |
| hemicurcumin (**1**) | 0.326655 | 0.100442 |
| 8’-β-apocarotenal | 0.934414 | 0.986177 |
| 12’-apo-β-carotenal | 0.421912 | 0.220204 |
| 8’-apo-β-astaxanthinal |  |  |
| 8’-apolycopenal | 0.799101 | 0.459658 |
| 12,12’-apo-dialdehyde |  |  |
| Crocetindial |  |  |
| zeaxanthin | 0.026995 | 0.368713 |
| β-chryptoxanthin | 0.757573 | 0.419195 |
| capsanthin | 0.287366 | 0.500596 |
| lutein | 0.436289 | 0.786228 |

| compound | etanol | PBS |
| --- | --- | --- |
| **2** | 0.539775 | 0.517354 |
| **3** | 0.580198 | 0.556527 |
| **4** | 0.033714 | 0.26687 |
| **5** | 0.458616 | 0.070297 |
| **6** | 0.024895 | -8.5E-16 |
| **7** | 0.323387 | 0.774496 |
| **8** | 0.648492 | 0.86848 |
| **9** | 0.825442 | 0.987206 |
| **10** | 0.865756 | 0.970124 |
| **11** | 0.858022 | 0.669063 |
| **12** | 0.765797 | 0.86875 |

**Table S2.** Effect of the solvent (comparison of the TEAC values measured in Ethanol and in PBS)

| compound | **F** | **homogeneity of variance** | **ANOVA** |  | **Eta-squared** |
| --- | --- | --- | --- | --- | --- |
| **2** | 154.497493 | 0.402406 | 0.000241 |  | 0.986502 |
| **3** | 61.335059 | 0.236327 | 0.001435 |  | 0.967556 |
| **4** | 0.202560 | 0.134486 | 0.675976 | * | 0.480854 |
| **5** | 192.545483 | 0.186049 | 0.000156 |  | 0.001147 |
| **6** | 16.473138 | 0.070744 | 0.015365 |  | 0.899609 |
| **7** | 427.988153 | 0.239143 | 0.000032 |  | 0.995027 |
| **8** | 115.975421 | 0.085180 | 0.000422 |  | 0.982206 |
| **9** | 77.078005 | 0.429469 | 0.000928 |  | 0.973777 |
| **10** | 399.236750 | 0.252238 | 0.000037 |  | 0.994673 |
| **11** | 230.069910 | 0.194119 | 0.000110 |  | 0.990840 |
| **12** | 2963.093970 | 0.857860 | 0.000001 |  | 0.999274 |

*There is no statistically significant difference between the TEAC values in ethanol and PBS.

**Table S3.** Effect of the modification (comparison of the TEAC values measured in ethanol)

|  |  | ethanol | | | |
| --- | --- | --- | --- | --- | --- |
| compound | **F** | **homogeneity of variance** | **ANOVA** | **Post Hoc** | **partial eta^2^** |
| **2** | 297.4951 | 0.303561 | 9.95E-07 | D | 0.990016 |
| **3** | 902.4051 | 0.163118 | 3.64E-08 | D | 0.996687 |
| **4** | 1653.184 | 0.025782 | 5.94E-09 | D | 0.998189 |
| **5** | 109.67 | 0.052673 | 0.000019 | D | 0.973374 |
| **6** | 1905.199 | 0.019093 | 3.89E-09 | D | 0.998428 |
| **7** | 2147.255 | 0.029988 | 2.72E-09 | D | 0.998605 |
| **8** | 393.5589 | 0.088413 | 4.33E-07 | D | 1 |
| **9** | 37.1854 | 0.164834 | 4.16E-04 | E | 0.925346 |
| **10** | 416.3652 | 0.107642 | 3.66E-07 | D | 0.992846 |
| **11** | 850.3274 | 0.080966 | 4.35E-08 | D | 0.996484 |
| **12** | 17396.85 | 0.826238 | 5.13E-12 | D | 0.999828 |

**Table S4.** Effect of the modification (comparison of the TEAC values measured in PBS)

|  | PBS | | | | |
| --- | --- | --- | --- | --- | --- |
| compound | **F** | **homogeneity of variance** | **ANOVA** | **Post Hoc** | **partial eta^2^** |
| **2** | 491.3467 | 0.121735 | 2.24E-07 | D | 0.993931 |
| **3** | 740.2614 | 0.051956 | 6.58E-08 | D | 0.995964 |
| **4** | 5108.41 | 0.021783 | 2.02E-10 | D | 0.999413 |
| **5** | 126.088 | 0.626756 | 0.000013 | D | 0.97676 |
| **6** | 9686.247 | 0.019964 | 2.97E-11 | D | 0.99969 |
| **7** | 10302.88 | 0.069153 | 2.47E-11 | D | 0.999709 |
| **8** | 20445.86 | 0.583433 | 3.16E-12 | D | 1 |
| **9** | 1642.436 | 0.575562 | 6.06E-09 | D | 0.998177 |
| **10** | 6751.415 | 0.869776 | 8.76E-11 | D | 0.999556 |
| **11** | 25785.25 | 0.127618 | 1.57E-12 | D | 0.999884 |
| **12** | 25260.92 | 0.064433 | 1.67E-12 | D | 0.999881 |

D: The compared groups are statistically significantly different.

E: There is no statistical difference between the measured TEAC values of the zeaxanthin and curcumin in ethanol.

**3. A shiny, dark crystal of curcumin-carotenoid hybrid**


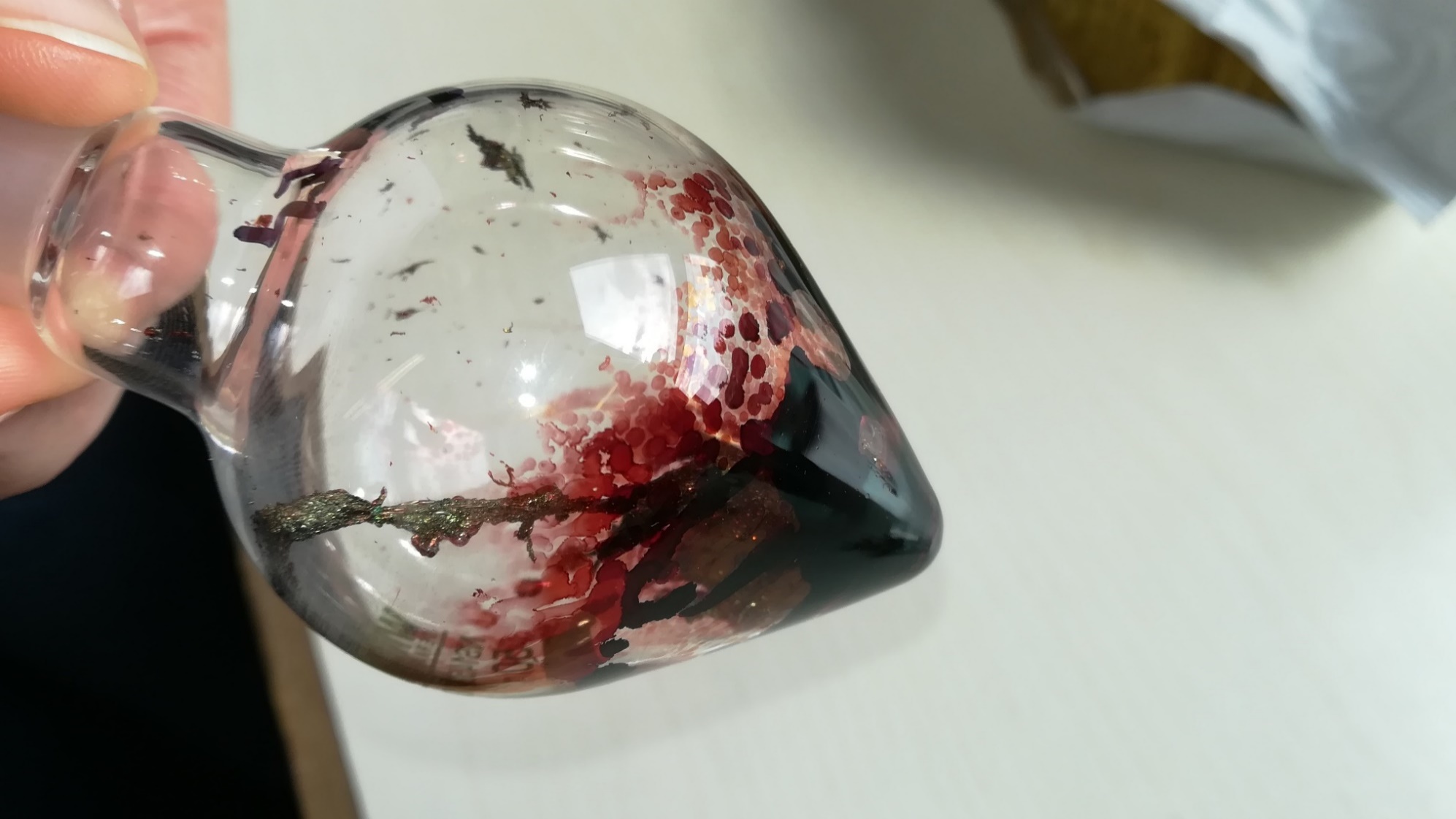


**Fig S6.** Crystals of 8’-apo-β-carotenal-hemicurcumin hybrid (**2**).
