# Supplementary material for: Synthesis, antioxidant capacity and aggregation of carotenoid-curcumin conjugates and hybrids
Source: PLoS One. 2026 May 11;21(5):e0347640. doi: 10.1371/journal.pone.0347640 (PMC13160358; doi:10.1371/journal.pone.0347640)
Supplement: S1 Appendix — (PDF) [file pone.0347640.s002.docx]

**Appendix - Experimental Section**

**Synthesis of hemicurcumin (1) (*E*)-6-(4-hydroxy-3-methoxyphenyl)hex-5-ene-2,4-dione)**

To 0.572 g (8.2 mmol) of B_2_O_3_ 2,4-pentanedione (1.11 mL, 10.6 mmol) was added and the mixture was stirred at room temperature for 18 hours under nitrogen (alternatively, the mixture can be heated with a heating mantle close to boiling and used immediately). Then 10.6 mL of dry ethyl acetate (EtOAc), 3.22 g of vanillin (21.2 mmol) and 11.66 mL of (BuO)_3_B (42.93 mmol) were added and stirring followed for 15 min. After that 0.212 mL of butylamine (2.12 mmol) was added slowly in 30 mins. Another stirring period followed for 18 hours. HCl (15.90 ml, 0.4 M) was added then heated to 60 ºC and stirring was continued for 1 hour. The resulting mixture was extracted with 50 mL of EtOAc. The combined organic phases were washed with brine, dried over MgSO_4_ and evaporated. The solid residue was triturated with methanol in a mortar, and put in the fridge to crystallize, resulting in 2.80 g (75%) yellow product. Mp. 148-150 °C. MS (MALDI-TOF) m/z 235 (M+H^+^). ^1^H NMR (500 MHz, CDCl_3_) δ (ppm): 2.15 (s, 3H, H-1), 3.94 (s, 3H, H-13), 5.63 (s, 1H, H-3), 5.90 (br s, 1H, OH), 6.32 (*d*, *J* = 15.8 Hz, 1H, H-5), 6.91 (*d*, *J* = 8.2 Hz, 1H, H-11), 7.01 (*d*, *J* = 1.8 Hz, 1H, H-8), 7.08 (*dd*, *J* = 8.2, 1.8 Hz, 1H, H-12), 7.53 (*d*, *J* = 15.8 Hz, 1H, H-6).

**General procedure for the condensation of hemicurcumin with apocarotenoid aldehydes**

1 eq. of hemicurcumin was dissolved in dry ethyl acetate (100 mg/mL) then 1.1 eq of B_2_O_3_ was added at 80 °C followed by the addition of 0.5 eq. of the aldehyde dissolved in dry ethyl acetate (50 mg/mL) and 1 eq. of (BuO)_3_B under argon. The solution was stirred for 30 mins, then piperidin (70 µL/L mmol hemicurcumin) was added and stirred for 30 mins at 80 ºC, and 0.4 M HCl (3 mL/1 mmol hemicurcumin) was added at 50 ºC and stirred for 30 mins, then cooled down in an ice bath. It was extracted with CH_2_Cl_2_ three times and washed with water and brine. Lastly the organic phase was dried and evaporated. For purification on silica gel with column chromatography and PLC hexane:acetone 7:3 and 6:4 mixtures were used. Yields are given at 80 % conversion of the aldehyde.

*8’-apo-β-carotenal hemicurcumin hybrid (****2****)*

Violet solid, yield 50%. M.p. 139 °C, λ_max_ (chloroform): 520, 420 nm. MS (MALDI-TOF) m/z 632.4 (M^+^). ^1^H NMR (500 MHz, CDCl_3_) δ (ppm): 1.03 (*s*, 6H, H-16, H-17), 1.46-1.48 (*m*, 2H, H-2), 1.61-1.63 (*m*, 2H, H-3), 1.72 (s, 3H, H-18), 1.99, 2.01 (2*s*, 12H, H-19,19’, H-20,20’), 2.02 (*m*, 2H, H-4), 3.95 (*s*, 3H, OCH_3_), 5.75 (*s*, 1H, H-3”), 5.84 (*brs*, 1H, OH), 6.07 (*d*, *J* = 15.3 Hz, 1H, H-1”), 6.11-6.21 (*m*, 3H, H-7, H-8, H-10), 6.26 (*d*, *J* = 11.3 Hz, 1H, H-12), 6.34-6.37 (*m*, 2H, H-14,14’), 6.46 (*d*, *J* = 15.8 Hz, 1H, H-5”), 6.53 (*d*, *J* = 14.4 Hz, 1H, H-12’), 6.55 (*d*, *J* = 8.2 Hz, 1H, H-10’), 6.56-6.74 (*m*, 4H, H-11,11’, H-15,15’), 6.93 (*d*, *J* = 8.1 Hz, 1H, H-11”), 7.05 (*s*, 1H, H-8”), 7.12 (*d*, *J* = 7.8 Hz, 1H, H-12”), 7.39 (*d*, *J* = 15.3 Hz, 1H, H-8’), 7.57 (*d*, *J* = 15.7 Hz, 1H, H-6”). ^13^C-NMR (125 MHz, CDCl_3_) δ (ppm): 12.65, 12.73, 12.8, 12.9 (C-19,19’ C-20,20’), 19.3 (C-3), 21.8 (C-18), 29.0 (C-16, C-17), 33.2 (C-4), 34.3 (C-1), 39.8 (C-2), 56.0 (OCH_3_), 101.4 (C-3”), 109.7 (C-8”), 114.9 (C-11”), 122.1 (C-5”), 122.4 (C-1”), 122.8 (C-12”), 124.2 (C-11’), 125.7 (C-11), 127.0 (C-7), 127.8 (C-7”), 129.5 (C-5), 129.6 (C-15’), 130.8 (C-10), 131.8 (C-15), 132.2 (C-12), 133.9 (C-9’), 135.3 (C-14’), 135.9 (C-13’), 136.5 (C-13), 137.0 (C-14), 137.69 (C-9), 137.73 (C-8), 138.0 (C-6), 140.0 (C-10’), 140.2 (C-6”), 142.0 (C-12’), 144.9 (C-8’), 146.8 (C-10”), 147.8 (C-9”), 183.0 (C-2”), 183.3 (C-4”). Analysis calc. for C_43_H_52_O_4_: C 81.61, H 8.28; found C 81.52, H 8.31.

*12’-apo-β-carotenal hemicurcumin hybrid (****3****)*

Violet solid, yield 45%. M.p. 90 °C, λ_max_ (chloroform): 500, 396 nm. MS (MALDI-TOF) m/z 568 (M+H^+^). ^1^H NMR (500 MHz, CDCl_3_) δ (ppm): 1.04 (*s*, 6H, H-16, H-17), 1.46-1.48 (*m*, 2H, H-2), 1.61-1.63 (*m*, 2H, H-3), 1.73 (*s*, 3H, H-18), 1.95, 1.98, 2.01 (3*s*, 9H, H-19, H-20,20’), 2.03 (*m*, 2H, H-4), 3.93 (*s*, 3H, O-CH_3_), 5.73 (*s*, 1H, H-3”), 6.06 (*d*, *J* = 15.4 Hz, 1H, H-1”), 6.10-6.23 (*m*, 3H, H-7, H-8, H-10), 6.26 (*d*, *J* = 11.8 Hz, 1H, H-12), 6.35 (*d*, *J* = 15.0 Hz, 1H, H-14), 6.45 (*d*, *J* = 15.8 Hz, 1H, H-5”), 6.55 (*d*, *J* = 12.1 Hz, 1H, H-14’), 6.59-6.64 (*m*, 1H, H-15’), 6.67-6.73 (*m*, 1H, H-15), 6.75-6.82 (*m*, 1H, H-11), 6.92 (*d*, *J* = 8.2 Hz, 1H, H-11”), 7.03 (*s*, 3H, H-8”), 7.10 (*d*, *J* = 8.1 Hz, 1H, H-12”), 7.37 (*d*, *J* = 15.3 Hz, 1H, H-12’), 7.56 (*d*, *J* = 15.7 Hz, 1H, H-6”). ^13^C NMR (125 MHz, CDCl_3_) δ (ppm): 12.5, 12.8, 12.9 (C-19, C-20,20’), 19.3 (C-3), 21.7 (C-18), 29.0 (C-16, C-17), 33.1 (C-4), 34.3 (C-1), 39.7 (C-2), 55.9 (OCH_3_), 101.5 (C-3”), 109.6 (C-8”), 114.8 (C-11”), 122.0 (C-5”), 122.5 (C-1”), 122.8 (C-12”), 126.4 (C-15), 127.3 (C-7), 127.7 (C-7”), 129.0 (C-15’), 129.6 (C-5), 130.6 (C-10), 131.8 (C-12), 133.9 (C-11), 134.2 (C-13’), 136.8 (C-14), 137.0 (C-9), 137.6 (C-8), 137.9 (C-6), 139.1 (C-13), 139.7 (C-14’), 140.3 (C-6”), 144.7 (C-12’), 146.8 (C-9”), 147.8 (C-10”), 182.8 (C-2”), 183.4 (C-4”). Analysis calc. for C_38_H_46_O_4_: C 80.53, H 8.18; found C 80.65, H 8.20.

*8’-apoastaxanthinal hemicurcumin hybrid (****4****)*

Deep violet solid, yield 44%. M.p. 188 °C, λ_max_ (chloroform): 514 nm. MS (MALDI-TOF) m/z 663 (M+H^+^), 685 (M+Na^+^). ^1^H NMR (500 MHz, DMSO) δ (ppm): 1.15 and 1.28 (2*s*, 6H, H-2a, H-16, H-17), 1.71 (*s*, 3H, H-18), 1.85 (*brs*, 1H, H-2_ax_), 1.98 (*s*, 12H, H-19,19’, H-20,20’), 2.08 (*brs*, H, H-2_eq_), 3.83 (*s*, 3H, OCH_3_), 4.19-4.22 (*m*, 1H, H-3), 5.04 (*s*, 1H, H-3”), 6.05 (*brs*, 1H, H-1”), 6.22-6.52 (*m*, 6H, H-7, H-8, H-10, H-12, H-14,14’), 6.62-6.83 (*m*, 8H, H-5”, H-11”, H-10’, H-11,11’, H-12’, H-15,15’), 7.14 (*d*, *J* = 7.2 Hz, 1H, H-12”), 7.31 (*brs*, 1H, H-8"), 7.34 (*d*, *J* = 15.7 Hz, 1H, H-8’), 7.54 (*d*, *J* = 15.9 Hz, 1H, H-6”). ^13^C-NMR (125 MHz, DMSO) δ (ppm): 12.2, 12.3, 12.4, 12.5 (C-19,19’, C-20,20’), 13.6 (C-18), 25.8, 30.2 (C-16, C-17), 36.4 (C-1), 45.8 (C-2), 55.6 (OCH_3_), 68.3 (C-3), 101.1 (C-3”), 111.2 (C-8”), 115.6 (C-11”), 121.2 (C-5”), 122.5 (C-1”), 123.0 (C-12”), 123.9, 124.8, 125.3 (C-7, C-11,11’), 126.2 (C-7”), 126.8 (C-5), 130.7, 131.6 (C-15,15’), 1334, 134.5, 135.0 (C-10, C-14,14’), 134.1, 135.0, 136.4, 137.0 (C-9,9’, C-13,13’), 138.9, 139.7 (C-10’, C-12), 140.7 (C-6”), 141.2, 141.7 (C-8, C-12’), 144.1 (C-8’), 147.9 (C-9”), 149.3 (C-10”), 159.7 (C-6), 181.8 (C-2”), 183.8 (C-4”), 199.7 (C-4). Analysis calc. for C_43_H_50_O_6_: C 77.92, H 7.60; found C 77.79, H 7.72.

*8’-apolycopenal hemicurcumin hybrid (****5****)*

Deep violet solid, yield 46%. M.p. 180 °C, λ_max_ (chloroform): 529, 427 nm. MS (MALDI-TOF) m/z 632.4 (M^+^). ^1^H NMR (500 MHz, CDCl_3_) δ (ppm): 1.62 (*s*, 3H, H-17), 1.69 (*s*, 3H, H-16), 1.82 (*s*, 3H, H-18), 2.04 (*m*, 12H, H-19,19’, H-20,20’), 2.03-2.16 (*m*, 4H, H-3, H-4), 3.95 (*s*, 3 H, OCH_3_), 5.11 (*br s*, 1H, H-2), 5.75 (*s*, 1H, H-3”), 5.96 (*d*, *J* = 10.4 Hz, 1H, H-6), 6.07 (*d*, *J* = 15.6 Hz, 1H, H-1”), 6.19 (*d*, *J* = 12.0 Hz, 1H, H-10), 6.24-6.31 (*m*, 2H, H-8, H-12), 6.34-6.38 (*m*, 2H, H-14,14’), 6.47 (*d*, *J* = 15.7 Hz, 1H, H-5”), 6.05-6.56 (*m*, 3H, H-7, H-10’, 12’), 6.61-6.74 (*m*, 4H, H-11,11’, H-15,15’), 6.93 (*d*, *J* = 7.9 Hz, 1H, H-11”), 7.05 (*s*, 1H, H-8”), 7.13 (*d*, *J* = 7.5 Hz, 1H, H-12”), 7.39 (*d*, *J* = 15.5 Hz, 1H, H-8’), 7.56 (*d*, *J* = 16.0 Hz, 1H, H-6”). ^13^C NMR (125 MHz, CDCl_3_) δ (ppm): 12.65, 12.74, 12.85, 12.93 (C-19,19’, C-20,20’), 17.0 (C-18), 17.7 (C-17), 25.7 (C-16), 26.7 (C-3), 40.3 (C-4), 56.0 (OMe), 101.5 (C-3”), 109.7 (C-8”), 114.8 (C-11”), 122.1 (C-5”), 122.4 (C-1”), 122.8 (C-12”), 123.9 (C-2), 124.2 (C-11’), 125.1 (C-7), 125.7 (C-6), 125.8 (C-11), 129.7 (C-15’), 131.4 (C-10), 131.8 (C-15), 132.4 (C-12), 133.9 (C-9’), 135.3 (C-8), 135.4 (C-14’), 136.0 (C-13’), 136.7 (C-9), 137.2 (C-14), 137.7 (C-13), 139.7 (C-5), 140.0 (C-10’), 140.3 (C-6”), 142.0 (C-12’), 144.9 (C-8’), 146.8 (C-9”), 147.8 (C-10”), 183.0, 183.3 (C-2”, C-4”). Analysis calc. for C_43_H_52_O_4_: C 81.61, H 8.28; found C 81.67, H 8.24.

*12,12’-diapo-dialdehyde semicurcumin hybrid (****6****)*

Red solid, yield 39%. M.p. >280 °C, λ_max_ (chloroform): 487nm. MS (MALDI-TOF) m/z 597 (M+H^+^). ^1^H NMR (500 MHz, DMSO-d_6_) δ (ppm): 2.00 (*s*, 6H, H-20,20’), 3.83 (*s*, 6H, 2x OCH_3_), 6.09 (*s*, 2H, 2x H-3”), 6.32 (*d*, *J* = 15.1 Hz, 2H, 2x H-1”), 6.61-6.87 (*m*, 2H, H-15,15’), 6.75 (*d*, *J* = 15.7 Hz, 2H, 2x H-5”), 6.82 (*d*, *J* = 7.9 Hz, 2H, 2x H-11”), 6.98-7.0 (*m*, 2H, H-14,14’), 7.15 (*d*, *J* = 7.3 Hz, 2H, 2x H-12”), 7.27-7.36 (*m*, 4H, 2x H-8”, H-12,12’), 7.56 (*d*, *J* = 15.6 Hz, 2H, 2x H-6”), 9.67 (*s*, 2H, 2x OH). ^13^C NMR (125 MHz, DMSO-d_6_) δ (ppm): 12.4 (C-20,20’), 55.6 (OMe), 101.3 (C-3”), 111.3 (C-8”), 115.6 (C-11”), 121.2 (C-5”), 123.1 (C-1”), 123.9 (C-12”), 126.1 (C-7”), 133.1 (C-15,15’), 136.4 (C-13,13’), 138.6 (C-14,14’), 141.1 (C-6”), 143.3 (C-12,12’), 147.9 (C-9”), 149.3 (C-10”), 181.0, 184.5 (C-2”, C-4”). Analysis calc. for C_36_H_36_O_8_: C 72.47, H 6.08; found C 72.68, H 5.95.

*Crocetindial semicurcumin hybrid (****7****)*

Deep violet solid, yield 30%, λ_max_ (chloroform): 497 nm. MS (MALDI-TOF) m/z 728.1 (M^+^). ^1^H NMR shows ca. 85% purity; ^1^H NMR (500 MHz, DMSO-d_6_) δ (ppm): 2.00 (*s*, 12H, H-19,19’, H-20,20’), 3.85 (*s*, 6H, 2x OCH_3_), 6.07 (*s*, 2H, 2x H-3”), 6.24 (*d*, *J* = 16.0 Hz, 2H, 2x H-1”), 6.56 (*brs*, 2H, H-14,14’), 6.71-6.80 (*m*, 4H, H-12,12’, H-15,15’), 6.77 (*d*, *J* = 15.0 Hz, 2H, 2x H-5”), 6.84 (*d*, *J* = 8.1 Hz, 2H, 2x H-11”), 7.06 (*d*, *J* = 14.7 Hz, 2H, H-10,10’), 7.17 (*d*, *J* = 7.5 Hz, 2H, 2x H-12”), 7.28-7.36 (*m*, 4H, 2x H-8”, H-8,8’), 7.56 (*d*, *J* = 15.9 Hz, 1H, H-6”). ^13^C NMR (125 MHz, DMSO-d_6_) δ (ppm): 13.0 (C-19,19’, C-20,20’), 56.2 (OMe), 101.3 (C-3”), 111.9 (C-8”), 116.2 (C-11”), 121.0 (C-5”), 123.0 (C-1”, C-12”), 124.6 (C-11, C-11’), 126.2 (C-7”), 131.3, 131.6 (C-14,14’, C-15,15’), 135.0 (C-9,9’), 139.7 (C-10,10’), 140.2 (C-13,13’), 140.5 (c-6”), 141.6 (C-12,12’), 144.1 (C-8,8’), 147.8 (C-9”), 149.3 (C-10”), 183.1 (C-4”, C-2”).

**General procedure for the synthesis of curcumin-carotenoid conjugates (8-12)**

30 mg of carotenoid succinate was dissolved in 2 mL of abs. DMF and 3 eq. (to bissucinates 6 eq.) of curcumin and 4 eq. (to bissuccinates 8 eq.) of DMAP was added. After 10 minutes 10 eq. of DCC was added. After 4 more hours additional 2 eq. of the curcumin and 10 eq. of DCC was added and the solution kept stirred overnight. After 20-24 hours of reaction time TLC showed no starting material and a less polar main product appeared. The mixture was poured into 100 mL diethyl ether and cooled down to precipitate dicyclohexyl carbamide. After filtration the etherial phase was washed three times with 50 mL of brine, dried and evaporated. Column chromatography on silica gel 60 proved to be insufficient for purification, so PLC was used instead using hexane: acetone 7:3 to 6:4 as eluent. The crude products were dissolved in small amounts of dichloromethane, and precipitated with hexane.

*8′-Apo-β-carotenol succinate monocurcumin conjugate* *(****8****)*

Orange crystals, yield: 70%. Mp: 115-116 °C. λ_max_ (chloroform): 410, 431 nm. MS (MALDI-TOF) *m/z* = 869 (M^+^). ^1^H NMR (500 MHz, CDCl_3_) δ (ppm): 1.03 (*s*, 6H, H-16, H-17), 1.47-1.48 (*m*, 2H, H-2), 1.61-1.62 (*m*, 2H, H-3), 1.72 (*s*, 3H, H-18), 1.84 (*s*, 3H, H-19’), 1.95, 1.96 (2 *s*, 12H, H-19, H-20,20′), 2.02 (*m*, 2H, H-4), 2.80 and 2.95 (2 *m*, 4H, 2 CH_2_-succinate), 3.86 and 3.94 (2 *s*, 6H, 2 OCH_3_), 4.61 (*s*, 2H, H-8′), 5.82 (*s*, 1H, H-1”), 5.89 (*brs*, 1H, OH), 6.11-6.24 (*m*, 6H, H-7, H-8, H-10,10’, H-14,14’), 6.33 (*d*, *J* = 14.8 Hz, 2H, H-12,12’), 6.42-6.67 (*m*, 6H, H-3”,3’”, H-11,11’, H-15,15’), 6.93 (*d*, *J* = 7.8 Hz, 1H, 7”’), 7.05-7.13 (*m*, 5H, H-6”,6”’, H-7”, H-10”,10”’), 7.59 and 7.60 (2 *d*, *J* = 15.4 Hz and 15.8 Hz, 2H, H-4”,4”’). ^13^C NMR (125 MHz, CDCl_3_) δ (ppm): 12.75, 12.77, 12.82 (C-19, C-20,20′), 14.8 (C-19′), 19.3 (C-3), 21.7 (C-18), 28.98 (C-16, C-17), 29.1, 29.3 (2 CH_2_-succinate), 33.1, 34.3 (C-4, C-1), 39.7 (C-2), 55.95, 55.97 (2 OCH_3_), 70.3 (C-8′), 101.5 (C-1”), 109.7, 111.5 (C-10”,10”’), 114.9 (C-7”’), 120.9, 121.8 (C-3”,3”’), 123.0, 123.29, 123.32 (C-11’, C-6”,6”’), 124.3 (C-7”), 125.3 (C-11), 126.7 (C-7), 127.6 (C-5”’), 129.3 (C-10’), 129.4 (C-5), 129.6, 130.5 (C-15,15’), 130.8 (C-10), 131.6 (C-9’), 132.2, 133.0 (C-14,14’), 134.1 (C-5”), 135.7, 136.1 (C-9, C-13’), 136.8 (C-13), 137.1 (C-12), 137.7 (C-8), 137.9 (C-6), 138.6 (C-12’), 139.4, 141.1 (C-4”,4”’), 141.1 (C-8”), 146.8, 148.0 (C-8”’, C-9”’), 151.4 (C-9”), 170.2 and 171.7 (2 C=O succinate), 181.8 and 184.5 (C-2”,2”’). Analysis calc. for C_55_H_64_O_9_: C 76.01, H 7.42; found C 76.10, H 7.38.

*Zeaxanthin bissuccinate biscurcumin conjugate* *(****9****)*

Orange crystals, yield: 81%,. mp: 116-117 °C. λ_max_ (in chloroform): 432, 458, 490 nm. MS (MALDI-TOF) *m/z* = 1469 (M^+^). ^1^H NMR (500 MHz, CDCl_3_) δ (ppm): 1.07, 1.11 (2 *s*, 12H, H-16,16’, H-17,17’), 1.57-1.62 (*m,* 2H, H-2_ax_, H-2′_ax_), 1.72 (*s*, 6H, H-18,18′); 1.79 (*m*, 2H, H-2_eq_, H-2′_eq_), 1.97 *(brs*, 12H, H-19,19′, H-20,20′), 2.13 (*dd*, *J* = 9.5 Hz, *J* = 16.6 Hz, 2H, H-4_ax_, H-4′_ax_), 2.45 (*dd*, *J* = 5.3 Hz, *J* = 16.9 Hz, 2H, H-4_eq_, H-4′_eq_), 2.75 and 2.94 (2 *m*, 8H, 2x2 CH_2_-succinate), 3.88 and 3.95 (2 *s*, 12H, 2x2 OCH_3_), 5.11 (*m*, 2H, H-3,3′), 5.83 (*s*, 2H, 2x H-1”), 6.07-6.12 (*m*, 4H, H-7,7’, H-8,8’), 6.16 (*d*, *J* = 11.4 Hz, 2H, H-10,10’), 6.26 (*d*, *J* = 6.9 Hz, 2H, H-14,14’), 6.37 (*d*, *J* = 14.8 Hz, 2H, H-12,12’), 6.45-6.67 (*m*, 8H, 2x H-3”,3”’, H-11,11’, H-15,15’), 6.93 (*d*, *J* = 8.0 Hz, 2H, 2x H-7”’), 7.06-7.17 (*m*, 10H, 2x H-6”,6”’, 2x H-7”, 2x H-10”,10”’), 7.59-7.62 (*m*, 4H, 2x H-4”,4”’). ^13^C NMR (125 MHz, CDCl_3_) δ (ppm): 12.7, 12.8 (C-19,19′, C-20,20′), 21.5 (C-18,18′), 28.5, 30.0 (C-16,16′, C-17,17′), 29.1, 29.6 (2x2 CH_2_-succinate), 36.7 (C-1,1’), 38.4 (C-4,4’), 44.0 (C-2,2’), 56.0 (2x2 OCH_3_), 69.0 (C-3,3′), 101.5 (2x C-1”), 109.7 (2x C-10”’), 111.5 (2x C-10”), 114.9 (2x C-7”’), 120.9, 121.8 (2x C-3”,3”’), 123.0, 123.2 (2x C-6”,6”’), 124.3 (2x C-7”), 124.9, 125.2 (C-7,7’, C-11,11’), 125.5 (C-5,5’), 127.6 (2x C-5”’), 130.1, 131.5 (C-10,10’,15,15’), 132.7 (C-14,14’), 134.1 (2x C-5”), 135.6, 136.5 (C-9,9’, C-13,13’), 137.9 (C-6,6’), 137.7, 138.7 (C-8,8’, C-12,12’), 139.4, 141.1 (2x C-4”,4”’), 141.2 (2x C-8”), 146.8, 148.0 (2x C-8”’, 2x C-9”’), 151.4 (2x C-9”), 170.3 and 171.6 (2x 2 C=O succinate), 181.8 and 184.5 (2x C-2”,2”’). Analysis calc. for C_90_H_100_O_18_: C 73.55, H 6.86; found C 73.49, H 6.89.

*β-Cryptoxanthin succinate curcumin conjugate (****10****)*

Red crystals, yield: 69%. Mp: 143-144 °C. λ_max_ (chloroform): 432, 459, 489 nm. MS (MALDI-TOF) *m/z* = 1003 (M^+^). ^1^H NMR (500 MHz, CDCl_3_) δ (ppm): 1.08, 1.11 (2 *s*, 12H, H-16,16’, H-17,17’), 1.46-1.47 (*m*, 2H, H-2’), 1.60 (*m,* 3H, H-2_ax_, H-3′), 1.72 (*s*, 6H, H-18,18′); 1.79-1.81 (*m*, 1H, H-2_eq_), 1.97 *(s*, 12H, H-19,19′, H-20,20′), 2.02 (*m*, 2H, H-4’), 2.11-2.17 (*m*, 1H, H-4_ax_), 2.43-2.48 (*m*, 1H, H-4_eq_), 2.75 and 2.94 (2 *m*, 8H, 2x2 CH_2_-succinate), 3.88 and 3.95 (2 *s*, 12H, 2x2 OCH_3_), 5.11 (*m*, 1H, H-3), 5.83 (*s*, 1H, H-1”), 5.93 (*brs*, 1H, OH), 6.07-6.19 (*m*, 6H, H-7,7’, H-8,8’, H-10,10’), 6.25 (*m*, 2H, H-14,14’), 6.35 and 6.37 (2 *d*, *J* = 14.4 Hz, *J* = 14.7 Hz, 2H, H-12,12’), 6.49 and 6.55 (2 *d*, *J* = 15.6 Hz, *J* = 15.9 Hz, 2H, H-3”,3”’), 6.62-6.68 (*m*, 4H, H-11,11’, H-15,15’), 6.94 (*d*, *J* = 8.0 Hz, 1H, H-7”’), 7.06-7.17 (*m*, 5H, H-6”,6”’, H-7”, H-10”,10”’), 7.61 (*d*, *J* = 15.3 Hz, 2H, H-4”, 4”’). ^13^C NMR (125 MHz, CDCl_3_) δ (ppm): 12.75, 12.76, 12.8, 12.82 (C-19,19′, C-20,20′), 19.3 (C-3’), 21.5 (C-18), 21.8 (C-18′), 28.5, 30.0 (C-16, C-17), 29.0 (C-16’, C-17’), 29.1, 29.6 (2 CH_2_-succinate), 33.1 (C-4’), 34.3 (C-1’), 36.7 (C-1), 38.4 (C-4), 39.7 (C-2’), 44.0 (C-2), 55.97, 55.98 (2 OCH_3_), 69.0 (C-3), 101.5 (C-1”), 109.7 (C-10”’), 111.5 (C-10”), 114.9 (C-7”’), 120.9, 121.8 (C-3”,3”’), 123.0, 123.3 (C-6”,6”’), 124.3 (C-7”), 124.8 (C-7), 125.1, 125.2 (C-11,11’), 125.5 (C-5), 126.7 (C-7’), 127.6 (C-5”’), 129.4 (C-5’), 129.9, 130.2, 131.8, 131.5 (C-10,10’,C-15,15’), 132.4 (C-14’), 132.7 (C-14), 134.1 (C-5”), 135.5, 136.1, 136.3, 136.6 (C-9,9’, C-13,13’), 137.2 (C-12’), 137.7, 137.8, 138.7 (C-8,8’, C-12), 137.9 (C-6,6’), 139.4, 141.1 (C-4”,4”’), 141.2 (C-8”), 146.8, 148.0 (C-8”’, C-9”’), 151.4 (C-9”), 170.3 and 171.6 (2 C=O succinate), 181.8 and 184.5 (C-2”,2”’). Analysis calc. for C_65_H_78_O_9_: C 77.81, H 7.84; found C 77.88, H 7.82.

*Capsanthin bissuccinate biscurcumin conjugate* *(****11****)*

Red crystals, yield: 45%. mp: 147–148 °C. λ_max_ (chloroform): 418, 482 nm. MS (MALDI-TOF) *m/z* = 1485 (M^+^). ^1^H NMR (500 MHz, CDCl_3_) δ (ppm): 0.86, 1.08 (2*s*, 6H, H-16, H-17), 1.11, 1.17 (2*s*, 6H, H-16′, H-17′), 1.31 (*s*, 3H, H-18′), 1.58-1.62 (*m*, 2H, H-2_ax_, H-4′_β_), 1.72 (*s*, 3H, H-18), 1.75–1.88 (*m*, 2H, H-2_eq_, H-2′_β_), 1.95, 1.97, 1.99 (3*s*, 12H, H-19,19′, H-20,20′), 2.05–2.13 (*m*, 2H, H-4_ax_, H-2′_α_), 2.45 (*dd*, *J* = 5.0 Hz, *J* = 17.2 Hz, 1H, H-4_eq_), 2.71-2,76, 2.90-2.95 (2*m*, 8H, 2x2 CH_2_-succinate), 2.99 (*dd*, *J* = 8.9 Hz, *J* = 14.7 Hz, 1H, H-4′_α_), 3.87, 3.95 (2*s*, 12H, 2x2 OCH_3_), 5.12 (*m*, 1H, H-3), 5.30 (*m*, 1H, H-3′), 5.83 (*s*, 2H, 2x H-1”), 5.90 (*brs*, 2H, 2x OH), 6.08-6.12 (*m*, 2H, H-7, H-8), 6.16 (*d*, *J* = 11.4 Hz, 1H, H-10), 6.26 (*d*, *J* = 11.2 Hz, 1H, H‑14), 6.35-6.40 (*m*, 2H, H-12, H-14′), 6.42 (d, *J* = 14.9 Hz, 1H, H-7′), 6.49 and 6.55 (2*d*, *J* = 15.7 Hz, *J* = 15.3 Hz, 4H, 2x H-3”, 2x H-3’”), 6.58-6.73 (*m*, 6H, H-10’, H-12’, H-11,11’, H-15,15’), 6.94 (*d*, *J* = 8.1 Hz, 2H, 2x H‑7”’), 7.06-7.18 (*m*, 10H, 2x H-6”, 2x H-6”’, 2x H-7”, 2x H-10”, 2x H-10”’), 7.34 (*d*, *J* = 15.0 Hz, 1H, H-8′), 7.60 and 7.61 (2*d*, *J* = 15.6 Hz, *J* = 15.7 Hz, 4H, 2x H-4”, 2x H-4”’). ^13^C NMR (125 MHz, CDCl_3_) δ (ppm): 12.73, 12.77, 12.84, 12.9 (C-19,19′, C-20,20′), 20.73 (C-18’), 21.5 (C-18), 24.8, 25.6 (C-16’, C-17’), 28.5, 30.0 (C-16, C-17), 29.05, 29.13, 29.50, 29.6 (2x2 CH_2_-succinate), 36.7 (C-1), 38.4 (C-4), 42.2 (C-4’), 43.7 (C-1’), 44.0 (C-2), 47.6 (C-2’), 56.0 (2x2 OCH_3_), 58.6 (C-5’), 69.0 (C-3), 74.2 (C-3’), 101.5 (2x C-1”), 109.7 (2x C-10”’), 111.5 (2x C-10”), 114.9 (2x C-7”’), 120.6 (C-7’), 120.9, 121.8 (2x C-3”, 2x C-3”’), 123.0, 123.2, 123.3, 124.1 (2x C-6”, 2x C-6”’, C-11, C-11’), 124.3 (2x C-7”), 125.5 (C-7), 125.6 (C-5), 127.6 (2x C-5”’), 129.7, 131.4, 131.7, 132.4, 135.3, 142.1 (C-10, C-12’, C-14,14’, C-15,15’), 133.6 (C-9’), 134.1 (C-5”), 135.9, 136.0, 137.6 (C-9, C-13,13’), 137.5 (C-12), 138.0 (C-6), 138.7 (C-8), 139.4, 141.1 (2x C-4”, 2x C-4”’), 140.9 (C-10’), 141.2 (2x C-8”), 146.8, 148.0 (2x C-8”’, 2x C-9”’), 151.37, 151.39 (2x C-9”), 170.26, 170.31, 171.6 (2x2 C=O succinate), 181.8, 184.5 (2x C-2”, 2x C-2”’), 202.5 (C-6’). Analysis calc. for C_90_H_100_O_19_: C 72.76, H 6.78; found C 72.71, H 6.82.

*Lutein bissuccinate biscurcumin conjugate* *(****12****)*

Orange crystals, yield: 73%. Mp: 122-123 °C. λ_max_ (chloroform): 426, 450, 484 nm. MS (MALDI-TOF) *m/z* = 1469 (M^+^). ^1^H NMR (500 MHz, CDCl_3_) δ (ppm): 0.87, 1.01 (2*s*, 6H, H-16’, H-17’), 1.08, 1.11 (2*s*, 6H, H-16, H-17), 1.47 (*d*, *J* = 14.5 Hz, 1H, H-2’_α_), 1.58-1.62 (*m*, 1H, H-2_ax_), 1.65 (*s*, 3H, H-18′), 1.72 (*s*, 3H, H-18), 1.80 (*d*, *J* = 12.2 Hz, 1H, H-2_eq_), 1.85 (*dd*, *J* = 5.8 Hz, *J* = 14.3 Hz, 1H, H-2’_β_), 1.91 (*s*, 3H, H-19’), 1.97 (*s*, 9H, H-19, H-20, H-20′), 2.13 (*dd*, *J* = 6.8 Hz, *J* = 17.2 Hz, 1H, H-4_ax_), 2.40 (*d*, *J* = 9.1 Hz, 1H, H-6’), 2.45 (*dd*, *J* = 4.2 Hz, *J* = 16.4 Hz, 1H, H-4_eq_), 2.75, 2.94 (2*m*, 8H, 2x2 CH_2_-succinate), 3.87, 3.95 (2*s*, 12H, 2x2 OCH_3_), 5.12 (*m*, 1H, H-3), 5.35-5.45 (*m*, 2H, H-3’, H-7′), 5.50 (*s*, 1H, H-4’), 5.83 (*s*, 2H, H-1”), 6.06-6.17 (*m*, 5H, H-7, H-8,8’, H-10,10’), 6.25 (*d*, *J* = 6.8 Hz, 2H, H-14,14’), 6.36 (*d*, *J* = 14.6 Hz, 2H, H-12,12’), 6.49 and 6.55 (2*d*, *J* = 15.7 Hz, *J* = 15.8 Hz, 4H, 2x H-3”,3”’), 6.58-6.67 (*m*, 4H, H-11,11’, H-15,15’), 6.93 (*d*, *J* = 8.0 Hz, 2H, 2x H-7”’), 7.05-7.16 (*m*, 10H, 2x H-6”,6”’, 2x H-7”, 2x H-10”,10”’), 7.60 (*d*, *J* = 15.0 Hz, 4H, 2x H-4”,4”’). ^13^C NMR (125 MHz, CDCl_3_) δ (ppm): 12.74, 12.80, 13.1 (C-19,19′, C-20,20′), 21.5 (C-18), 23.0 (C-18′), 25.6, 28.9 (C-16’, C-17’), 28.5, 30.0 (C-16, C-17), 29.1, 29.3, 29.5, 29.7 (2x2 CH_2_-succinate), 33.3 (C-1’), 36.7 (C-1), 38.4 (C-4), 39.3 (C-2′), 44.0 (C-2), 55.0 (C-6’), 56.0 (2x2 OCH_3_), 69.0 (C‑3), 69.4 (C-3′), 101.4 (2x C-1”), 109.7 (2x C-10”’), 111.5 (2x C-10”), 114.9 (2x C-7”’), 119.7 (C-4’), 120.9, 121.8 (2x C-3”, 2x C-3”’), 123.0, 123.3 (2x C-6”, 2x C-6”’), 124.3 (2x C-7”), 124.8 (C-11’), 124.9 (C-11), 125.2 (C-7), 125.5 (C-5), 127.6 (2x C-5”’), 128.2 (C-7’), 130.07, 130.11 (C-15,15’), 131.0 (C-10’), 131.5 (C-10), 132.6 (C-14,14’), 134.1 (2x C-5”), 135.0 (C-9’), 135.6 (C-9), 136.4, 136.5 (C-13,13’), 137.7 (C-8’, C-12,12’), 137.9 (C-6), 138.7 (C-8), 139.4, 141.1 (2x C-4”, 2x C-4”’), 140.7 (C-5’), 141.2 (2x C-8”), 146.8, 148.1 (2x C-8”’, 2x C-9”’), 151.4 (2x C-9”), 170.28, 170.31, 171.6 (2x2 C=O succinate), 181.8, 184.5 (2x C-2”, 2x C-2”’). Analysis calc. for C_90_H_100_O_18_: C 73.55, H 6.86; found C 73.41, H 6.93.
